# A Multimodal Scaffold for SDF1 Delivery Improves Cardiac Function in a Rat Subacute Myocardial Infarct Model

**DOI:** 10.1021/acsami.3c04245

**Published:** 2023-08-11

**Authors:** Iñigo Perez-Estenaga, Merari Tumin Chevalier, Estefania Peña, Gloria Abizanda, Amir M. Alsharabasy, Eduardo Larequi, Myriam Cilla, Marta M. Perez, Jon Gurtubay, Manuel Garcia-Yebenes Castro, Felipe Prosper, Abhay Pandit, Beatriz Pelacho

**Affiliations:** † Regenerative Medicine Department, Center for Applied Medical Research (CIMA), University of Navarra, Pamplona 31008, Spain; ‡ CÚRAM, SFI Research Center for Medical Devices, University of Galway, Galway H91 TK33, Ireland; § Aragon Institute of Engineering Research, University of Zaragoza, Zaragoza 50009, Spain; ∥ CIBER-BBN—Centro de Investigación Biomédica en Red en Bioingeniería Biomateriales y Nanomedicina, Zaragoza 50018, Spain; ⊥ Instituto de Investigación Sanitaria de Navarra (IdiSNA), Pamplona 31009, Spain; ∇ Department of Anatomy, Embryology and Animal Genetics, University of Zaragoza, Zaragoza 50009, Spain; ○ Department of Cardiology, Clínica Universidad de Navarra, Madrid 28027, Spain; ◆ Department of Cell Therapy and Hematology, Clínica Universidad de Navarra, Pamplona 31008, Spain; ¶ CIBERONC, Madrid 28029, Spain

**Keywords:** Collagen scaffold, SDF1, Myocardial infarction, Biocompatibility, Angiogenesis

## Abstract

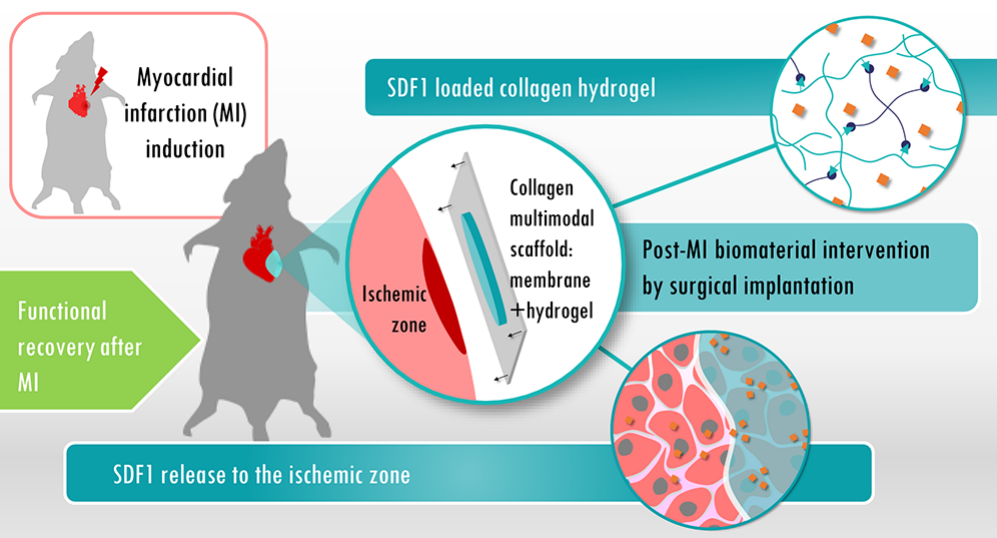

Ischemic heart disease is one of the leading causes of death worldwide. The efficient delivery of therapeutic growth factors could counteract the adverse prognosis of post-myocardial infarction (post-MI). In this study, a collagen hydrogel that is able to load and appropriately deliver pro-angiogenic stromal cell-derived factor 1 (SDF1) was physically coupled with a compact collagen membrane in order to provide the suture strength required for surgical implantation. This bilayer collagen-on-collagen scaffold (bCS) showed the suitable physicochemical properties that are needed for efficient implantation, and the scaffold was able to deliver therapeutic growth factors after MI. *In vitro* collagen matrix biodegradation led to a sustained SDF1 release and a lack of cytotoxicity in the relevant cell cultures. *In vivo* intervention in a rat subacute MI model resulted in the full integration of the scaffold into the heart after implantation and biocompatibility with the tissue, with a prevalence of anti-inflammatory and pro-angiogenic macrophages, as well as evidence of revascularization and improved cardiac function after 60 days. Moreover, the beneficial effect of the released SDF1 on heart remodeling was confirmed by a significant reduction in cardiac tissue stiffness. Our findings demonstrate that this multimodal scaffold is a desirable matrix that can be used as a drug delivery system and a scaffolding material to promote functional recovery after MI.

## 1 Introduction

Cardiovascular diseases (CVDs) are responsible for enormous health and economic burdens. With around 18 million lives lost yearly, these diseases are the leading cause of mortality worldwide. Among all CVDs, ischemic heart disease accounts for the most fatalities, representing 16% of all global deaths. In Europe alone, cardiac events, mainly myocardial infarction (MI), are responsible for 4 million deaths per year.^1^ MI is caused by a sudden, severe blockage of blood flow to the heart, which leads to cell death, an acute inflammatory response, and abnormal remodeling of the extracellular matrix with fibrotic scar formation.^2^ Therapeutic alternatives based on growth factors (GFs) and/or stem cells, which promote the regeneration of the injured heart, have been exhaustively studied.^3,4^ Many experimental MI preclinical models have shown the therapeutic benefit of pro-angiogenic, prosurvival, and/or antifibrotic GFs. However, the requirement for high doses and frequent administration of GFs in order to reach efficacy diminish their safety and benefits for ischemic patients.^3^

Extracellular matrix (ECM)-inspired, tunable biomaterials have been successfully designed as drug and/or cell delivery systems to treat MI.^5^ Collagens are the most abundant family among the macromolecules constituting ECMs and have been widely studied for therapeutic applications. Although almost 30 collagen types exist, collagen type I is the most frequently used in biomaterials development.^6,7^ We have previously reported on suturable collagen-I sheets as scaffolds for mesenchymal stem cell delivery, significantly improving their graft and survival into the ischemic cardiac tissue and, thus, their therapeutic effect. Importantly, the high compatibility of our collagen membrane (CM) with the cardiac tissue was shown.^8,9^ However, the properties of our fabricated collagen constructs were intrinsically insufficient for drug delivery applications. There are strategies to improve these properties (involving cross-linking) that are widely reported.^10,11^ Collagen bears primary ε-amino groups from their lysine residues^12^ that are reactive toward isothiocyanates, isocyanates, *N*-hydroxysuccinimide (NHS) esters, pentafluoroesters, aldehydes, or activated carboxyl groups.^13,14^ NHS esters are commonly introduced for cross-linking through the amidation of terminal amino groups; therefore, a wide variety of already NHS-functionalized molecules are commercially available on the market, extending the possible modifications of collagen substrates.^15^ Collagen hydrogels (CH) that are fabricated using this synthetic route can efficiently deliver hydrophilic active ingredients, as the hydrophilic polymer chains can accommodate and entrap water-miscible molecules once cross-linked.

The expression of stromal cell-derived factor 1 (SDF1), a potent chemoattractant that is also named CXCL12, is acutely increased in the heart during the first days after the infarct, thus favoring the recruitment of bone marrow vascular progenitor cells that revascularize the ischemic heart.^16,17^ However, this transient expression is insufficient for tissue repair, and a sustained exogenous expression is key for cardiac repair. Through their gradual biodegradation due to their short half-life in the *in vivo* micro-environment, covalently cross-linked, CH-carrying SDF1 can address this need.

In this study, we developed a multimodal bilayer collagen-on-collagen scaffold (bCS), combining the non-cross-linked, ultrathin CM with an SDF1-loaded collagen hydrogel (CH-SDF1), as a therapeutic candidate to modulate the angiogenic response post-MI. The rationale for the design of this scaffold was to develop a scaffold that had the required suturability and the desired therapeutic potential with a clinical translational potential.

## 2 Materials and Methods

### 2.1 Materials

The materials used in this study are as follows: bovine type I collagen (lyophilized and in sheets, Naturin/Viscofan S.A.), four-arm polyethylene glycol (4-arm PEG) succinimidyl glutarate (also known as 4S-StarPEG, JenKem Technology, USA, 10 000 g/mol), stromal cell-derived factor 1 (SDF1, Raybiotech), 1M NaOH, phosphate-buffered saline (PBS 10X and PBS 1X), and bacterial collagenase type IV from *Clostridium histolyticum* (Sigma-Aldrich, ≥125 CDU/mg).

### 2.2 Methods

#### 2.2.1 Fabrication of the SDF1-Loaded Type I Collagen Hydrogels (CH-SDF1)

The hydrogels were fabricated through covalent cross-linking between the intrinsically present collagen primary amino groups and the NHS ester reactive groups from a 4-arm PEG succinimidyl glutarate cross-linker. Briefly, for obtaining different concentrations of hydrogels, the appropriate amount of type I collagen was mixed with PBS 10X. Then, the resulting mixture was neutralized by adding a 1 M NaOH solution and kept in ice prior to gel formation by the addition of the stoichiometric amount of 4arm-PEG succinimidyl glutarate dissolved in PBS 1X. Next, 200 μL of the reaction mixture solution was pipetted and placed on a Teflon-coated slide to allow cross-linking for 1 h at 37 °C incubation. For gel cytokine loading, 100 ng of SDF1 (1 μL of a 100 μg/mL solution) was added to the mix before the addition of the cross-linker.

#### 2.2.2 Rheological Measurements

Rheological measurements were performed using a rotational rheometer (MCR 302, Anton Paar, Austria) with a parallel plate at 37 °C. Cylindrically swollen 3, 5, 6, and 6.5 mg/mL CH were fabricated and placed between the nonporous stainless steel parallel plates (diameter = 10 mm). The frequency sweep was tested between 0.01 and 100 Hz at a fixed strain corresponding to the hydrogel’s linear viscoelastic region. This was performed to further report the storage and loss moduli at 1 Hz. The mechanical properties of the bilayer scaffold that was prepared with the 5 mg/mL collagen hydrogel were also measured.

#### 2.2.3 Morphology of the Collagen Hydrogels and Porosity Assessment

The following equation was employed to estimate the porosity of the hydrogels:
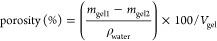
1

Here, *m*_gel1_ and *m*_gel2_ are the weight of the swollen (for 24 h) and freeze-dried CH, respectively, ρ_water_ is the density of pure water, and *V*_gel_ is the final hydrogel volume (200 μL). Any excess surface water was removed with filter paper before each measurement.

The morphology and microstructure of the collagen hydrogels were qualitatively assessed by field emission scanning electron microscopy (FESEM, Hitachi SU 8000 TED). Briefly, the fabricated CH were hydrated for 24 h and subsequently snap-frozen using liquid nitrogen, fractured, frozen at −80 °C, and finally freeze-dried. Images were obtained using a Hitachi S-4700 electronic microscope after the coating procedures. Pore size quantifications were performed by employing the Fiji (ImageJ) software package.

#### 2.2.4 Efficiency of Cross-Linking

Residual primary amine groups of the CH were determined using a 2,4,6-trinitrobenzenesulfonic acid (TNBSA) assay. In brief, after cross-linking and hydrogel formation, the CH were incubated in a 0.1 M sodium bicarbonate (pH = 8.5) solution. Then, 0.01% TNBSA was added to the samples and the mixture was incubated for 2 h at 37 °C. The reaction was stopped using 10% sodium dodecyl sulfate and 1 M hydrochloric acid (HCl). The samples were then incubated at 120 °C for 15 min. The absorbance of each sample was read at 335 nm, and the free amine groups were quantified by interpolating the values from a standard linear curve of known glycine concentrations. The cross-linking efficiency (CE) was calculated as follows:
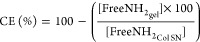
2

#### 2.2.5 *In Vitro* Biodegradation Studies

The degradation profile of the fabricated CH was evaluated *in vitro*, with the aim of estimating the SDF1 release from the polymer matrix. This correlates with the cleavage of the polymeric collagen chains that form the interconnected network. The hydrogels were incubated at 37 °C in the presence of 25, 50, and 100 ng/mL bacterial collagenase type IV for 5 days. The wet collagen hydrogel mass was measured at different fixed time points (1, 6, 12, 24, 48, 72, 96, and 120 h) using a Mettler Toledo AX26 DeltaRange scale.

#### 2.2.6 *In Vitro* SDF1 Release Assay

First, 5 mg of collagen/mL of CH-SDF1 was incubated at 37 °C in 25 ng/mL bacterial collagenase type IV or PBS alone. The samples were collected at 24, 48, 72, 96, and 120 h. Tubes containing the hydrogels were replenished with the same volume of the incubation medium after withdrawal. The release of SDF1 was quantified with an ELISA kit (RayBiotech).

#### 2.2.7 Cell Culture

Human adipose-derived mesenchymal stem cells (ADSC) were prepared by 3P Biopharmaceuticals (Pamplona, Spain) under Good Manufacturing Practice (GMP) conditions. The cells were cultured (5% CO_2_ and a humidified atmosphere) at a density of 7500 cells/cm^2^ in an ADSC medium (α-MEM, Gibco) supplemented with 10% fetal bovine serum (FBS, Biochrom), 1% penicillin/streptomycin (P/S, Lonza), and 1 ng/mL basic fibroblast growth factor (bFGF, Sigma-Aldrich).

A murine HL1 cardiac cell line (Sigma-Aldrich) was cultured under the same conditions at a density of 25 000 cells/cm^2^ in 0.1% gelatin-coated flasks and a Claycomb medium (Sigma-Aldrich) that contained 10% FBS and 1% P/S.

#### 2.2.8 *In Vitro* Hydrogel Cytotoxicity Assay

The cytotoxicity of the CH over HL1 and ADSC was studied by assessing the metabolic activity of the cells in the hydrogels in the presence of alamarBlue (Invitrogen, USA). First, 50 × 10^5^ ADSC or HL1 cells were seeded in a 48-well plate on 500 μL of culture medium and co-cultured after 12 h with 5 mg/mL CH. The controls consisted of cells that were cultured alone in the same conditions. After 72 and 168 h of incubation, the medium was withdrawn and substituted with 500 μL of working solution of alamarBlue (10% v/v in ADSC medium) and left at 37 °C. After 3 h, a volume of 100 μL was measured on a SPECTROstar Nano microplate reader (BMG Labtech, 570 nm emission and 600 nm excitation). The metabolic activity values of the cells in the presence of the hydrogels were normalized against the controls.

#### 2.2.9 *In Vitro* Bioactivity of Released SDF1 from CH

An *in vitro* migration assay with human umbilical vein endothelial cells (HUVEC) that were cultured in the presence of SDF1-loaded hydrogel supernatants was performed to determine the bioactivity of the hydrogel-released cytokine. In brief, the endothelial cells were cultured in complete medium (CoM) of MEM199 (Gibco), supplemented with 10% FBS, 1% P/S, ECGS (7.5 mg/mL, Sigma-Aldrich), and 10 units/mL heparin (Medical Iberica S.A.), and incubated at 37 °C. When the cells reached 95% confluency, the culture medium was replaced by a “HUVEC serum-free medium” (SFM; MEM199, 1% FBS, 10 units/mL heparin, and 1% P/S) and incubated at 37 °C for 12 h. Afterward, 4 × 10^4^ of the cells were seeded in a 24-well transwell (8 μm pore, CoStar) in 100 μL of SFM. Then, a 600 μL aliquot of stimulation medium (SFM, MEM199, 1% FBS, 1% P/S, and 20 ng/mL SDF1) was added to the lower chamber, and 600 μL of H-SFM or H-CM was added for negative and positive controls. In the experimental groups, supernatants of the digested hydrogels were diluted in SFM to give a final SDF1 concentration of 20 ng/mL before being added to the lower chamber of the transwells. After 8 h of cell migration, the nonmigrated cells were removed from the upper chamber with a swab and the membranes were fixed in methanol for 5 min. After washing, the membranes were stained with Harris Hematoxylin (Sigma-Aldrich, 1:2 dilution) for 8 min. Finally, after one more washing, the membranes were left to dry and mounted in DPX for cell counting via optical microscopy.

#### 2.2.10 Assembly of the Multimodal Bilayer Collagen-on-Collagen Scaffold

Two collagen-processing alternatives that render two different collagen-based materials were combined to provide a final bilayer collagen-on-collagen scaffold (termed bCS; termed bCS-SDF1 when loaded with the cytokine) that was able to fulfill the prerequirements of the proposed therapeutic biomaterial intervention. Briefly, a frame was used to assemble a 75 μL disc-shaped (SDF1 loaded or not) CH, which was then adhered to a 1.3 × 1.3 cm^2^ CM. The hydrogel reaction mixture was prepared and placed into the frame holding the CM and incubated for 1 h at 37 °C to allow cross-linking. Next, the frame was removed, and the bCS was maintained in a humidified, sterile container until implantation.

#### 2.2.11 The MI Rat Experimental Model and bCS Implantation

MI was induced in 10- to 12-week-old female Sprague Dawley rats (Envigo, average weight = 220 g) by permanent ligation of the left anterior descending (LAD) coronary artery, as has been previously described.^8^ Briefly, the rats were anesthetized with 4% isoflurane using an induction chamber, intubated, and ventilated at 90 cycles/min. Then, a left thoracotomy was performed through the fourth intercostal space to access the heart, and the LAD coronary artery occluded 2–3 mm distal from its origin. After a lack of blood irrigation was confirmed, the chest was closed in layers, and the rats were allowed to recover on a heating pad.

One week after infarction, the multimodal scaffolds were implanted into the hearts by suturing them at four points with a Prolene 7-0 suture (W8702, Ethicon) covering the infarcted area. The infarcted rats were implanted with either the bCS that was loaded with 10 μg of SDF1 (termed bCS-SDF1) or the unloaded bCS. Another group was infarcted but not implanted to act as the control group.

All animal procedures were approved by the University of Navarra Institutional Committee on Care and Use of Laboratory Animals and the European Community Council Directive, Ref 86/609/EEC.

#### 2.2.12 Cardiac Function Assessment by Echocardiography

Echocardiographic studies were performed 5 days post-infarct (2 days before patch implantation) and 2 months post-implantation. The animals with an ejection fraction (EF) below 35% were included in the functional study and were randomly distributed in the experimental groups. Images were acquired using a high-resolution Vevo 3100 imaging system, coupled to an MX550D linear-frequency transducer (central frequency of 40 MHz), with an axial resolution of 40 μm and a 14.6 mm field of view (FUJIFILM VisualSonics). Images were acquired at a frame rate of ∼200 frames per second. Briefly, the rats were anesthetized by inhalation of 2% isoflurane in 80% oxygen and placed on a handling platform in a supine position. Electrocardiography and respiratory rates were monitored using probes that were connected to the limbs of the animals. For the measurement of left ventricular function, end-systolic volumes (ESV) and end-diastolic volumes (EDV), as well as EF after MI, were determined by following the biplane Simpson method. An investigator, blinded to the treatment groups, acquired several Bright (B)-mode movies of the PSLAX and three orthogonal SAX regions (midventricular, apical, and basal). Two-dimensional B-mode movies were analyzed using VevoLab software (FUJIFILM VisualSonics) and the corresponding Simpson tools in the cardiac package. The endocardial cavity of the left ventricle from the three SAX B-mode regions (midventricular, apical, and basal) was manually traced at both the diastolic and systolic phases. In addition, the diastolic and systolic lengths of the left ventricle, from the mid mitral annulus to the cardiac apex, were obtained from the PSLAX B-mode. Using these measurements, the ESV (μL), EDV (μL), and EF (%) values were obtained. The software also generated the fractional shortening (FS, %) parameter values.

#### 2.2.13 Histological Analysis

The animals were perfusion-fixed and euthanized 7 and 30 days after implantation for biocompatibility analysis and 2 months post-implant for assessment of cardiac tissue revascularization. After sacrifice at the given time points, heart tissues were processed for histological analysis following standard protocols and sectioned in three serial sections of 10 slides, each measuring 5 μm in thickness.

Hematoxylin and eosin (H&E) staining was performed in order to assess inflammatory tissue reaction toward the patch. Briefly, heart sections were stained with Harris Hematoxylin (Merck) for 7 min, differentiated through 37% HCl–water and Li_2_CO_3_ saturated solutions, immersed in 0.5% eosin (Merck) for 10 s, dehydrated, and mounted in DPX. Additionally, macrophage infiltration and the M1/M2 phenotype were quantified in the implantation zones. Cell detection for the M1 macrophages was performed using a mix of mouse antibody CD68 (Bio-Rad) and rabbit antibody CCR7 (Abcam), both of which were diluted 1:100. For the M2 macrophage staining, the antibodies CD68 and CD206 (Abcam) were used, also diluted 1:100. Alexa Fluor 488 donkey anti-mouse IgG (Invitrogen) and Alexa Fluor 594 donkey anti-rabbit (Invitrogen), both diluted 1:200, were used as secondary antibodies. Collagen patch integration and degradation were assessed in sections stained with Sirius red (SR). Slides were deparaffinized and immersed in 0.1% Fast Red (Sigma-Aldrich) in a saturated solution of picric acid for 90 min, differentiated for 2 min in 0.01 M HCl (Sigma-Aldrich), dehydrated, and mounted in DPX (Sigma-Aldrich). Additionally, tissue revascularization was quantified by lectin and smooth muscle actin (SMA) stainings. After endogenous peroxidase saturation by 3% H_2_O_2_ (Sigma-Aldrich) for 20 min and antigen blocking with 1% bovine serum albumin (BSA, Sigma-Aldrich) in PBS for 30 min, the sections were incubated overnight at 4 °C with *Bandeiraea simplicifolia* (BS) lectin I (Sigma-Aldrich, diluted 1:50 in 1% BSA). After rinsing in PBS, the samples were incubated with a streptavidin–biotin complex (DAKO, diluted 1:75) for 45 min, developed with 3,3′-diaminobenzidine (DAKO) for 2 min, dehydrated, and mounted in DPX. For SMA immunofluorescence staining, sections were incubated overnight at 4 °C with the α-smooth muscle actin-Cy3 antibody (1:500 dilution, Sigma-Aldrich). After being rinsed in PBS, the samples were mounted in a DAPI-glycerol medium. Finally, lymphocyte and macrophage infiltration in the cardiac tissue was identified by immunohistochemistry staining, as previously described. Rabbit antibody CD3 (Abcam) and mouse antibody CD68 (Bio-Rad) were diluted 1:100 in PBS and incubated overnight.

#### 2.2.14 Morphometric Analysis

H&E, SR, lectin, and inflammatory cell infiltration pictures were captured at 20× magnification using an Aperio scanner (Scanner CS2, Leica Biosystems), and the stained tissues and positive cells were quantified using ImageJ (Fiji) software. The inflammatory heart areas were measured in the H&E pictures based on the intense purple color staining, which is due to the high nuclear density of the inflammatory cells. Two images of the zone surrounding the implant were taken per section in three serial sections. Pictures of SMA+ vessels and M1 and M2 macrophages at the implant infarct zone were acquired with an MR3 Axiocam camera (ZEISS) that had been adapted with the M1 Axio Imager ZEISS microscope. The number of capillaries/mm^2^ was counted on three serial lectin-stained sections and calculated as the mean number of 5–10 μm diameter vessels. The number of arterioles, arteries/mm^2^, and infiltrating lymphocytes and macrophages were counted on three serial CD3+- and CD68+-stained sections. Morphometric analysis was performed in three rats per group for histocompatibility analyses and 7–10 rats per group for vascular response studies.

#### 2.2.15 Mechanical Analysis

For mechanical analysis, rat heart tissue samples (15 mm × 3 mm) were taken from the infarct area near the coronary bifurcation and aligned longitudinally with the axial axis of the heart. Three measurements at different locations were taken using a Mitutoyo Digimatic micrometer, which held the measurement when the contact force reached a value of 0.5 N, to measure the samples’ length, width, and thickness. Simple tension tests were performed in a high-precision drive Instron MicroTester 5548 system using a 10 N load cell with a minimal resolution of 0.005 N. The strain was measured by a noncontact Instron 2663-281 video-extensometer. An ultrasonic humidifier was used to avoid specimen drying, loaded at a 1 mm/min displacement rate up to rupture. The elastic modulus at small strains (*E*_I_) and the tangent modulus before bond rupture (*E*_II_, named as tangent moduli) were computed and used as parameters to compare the mechanical properties of the different samples.^8^

#### 2.2.16 Statistical Analysis

Statistical analyses were performed with GraphPad Prism software (version 8.4.2). The samples’ normality was assessed using the Shapiro–Wilk and Kolmogorov–Smirnov normality tests. For samples that followed normal distributions, *t* tests or paired samples conventional *t* tests were used to compare two groups of independent samples; the Mann–Whitney U test was used for non-normal distributions. A one-way analysis of variance (ANOVA) test, followed by Sidak, was used for samples that had a normal distribution when three or more experimental groups were analyzed. For comparisons among experimental groups with samples with non-normal distributions, the Kruskal–Wallis one-way ANOVA test was employed, with multiple comparisons using Dunn’s test. Statistical significance was determined by *P* values: *P < 0.05, **P < 0.01, ***P < 0.001, and ****P < 0.0001.

## 3 Results and Discussion

### 3.1 Fabrication and Fundamental Characterization of CH and CH-SDF1

**Figure 1:**
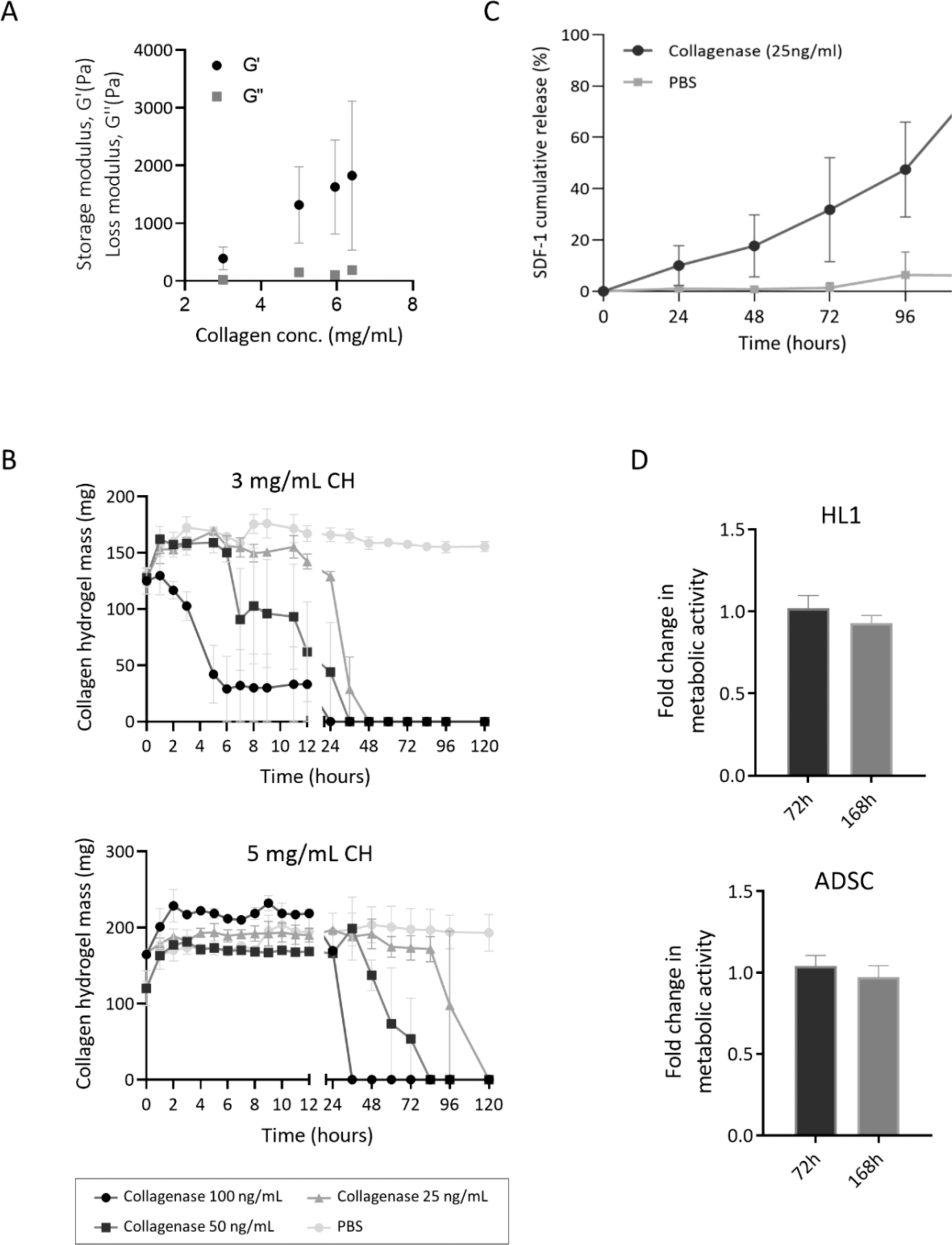
Screening and fundamental characterization of the collagen type I hydrogels. (A) A rheological assessment: the hydrogels’ storage modulus (*G*′) increases with final collagen concentration at stoichiometric amounts of the cross-linker. *G*′′ < *G*′ confirms the gel-like properties. (B) The biodegradation profile of the 3 and 5 mg/mL hydrogels in the presence of different concentrations of collagenase type IV. See the key below the graphs for details about the data. (C) An *in vitro* assessment of the SDF1 release profile for the 5 mg collagen/mL hydrogel. Hydrogels were incubated with 25 ng/mL collagenase for 120 h. (D) An *in vitro* assessment of the cytotoxicity of the 5 mg collagen/mL hydrogel over HL1 cardiac cells and ADSC. The fold changes in the metabolic activity of the cells that were cultured in the presence of the CH versus those cultured without CH are represented. A noncytotoxic profile was confirmed after 72 and 168 h of incubation, as no significant differences were found between the cells cultured with CH and those cultured without CH. The data are represented as mean ± SEM (*N* = 3).

CH with different concentrations of collagen type I content were successfully fabricated through covalent cross-linking by amidation and were fully characterized in order to assess their suitability for the proposed application (Figure S1A). Rheological measurements, which determined the storage (*G*′) and loss (*G*″) moduli, were performed to confirm the gel-like properties of the CH. The storage modulus corresponds to the elastic-solid-like behavior, and the loss modulus relates to the viscous portion. Having a storage modulus that is larger than the loss modulus indicates that elastic behavior governs the properties of the material.^15^ Different final collagen concentrations (3, 5, 6, and 6.5 mg/mL) in the hydrogels were initially tested to explore the mechanical properties, especially in terms of the storage modulus *G*′ (1 Hz, 37 °C). As mentioned above, the storage modulus reflects the hydrogel’s ability to store deformation energy in an elastic manner. However, *G*′ values in the linear viscoelastic region indicate the gel strength that is provided by the cross-linking of the polymer chains. When engineering a therapeutic biomaterial to treat MI, achieving non-immunogenic and functional properties that resemble the natural myocardium is of paramount importance.^18^ Our 3 and 5 mg/mL CH exhibited storage moduli of 393 ± 48 and 1319 ± 426 Pa, respectively, at the oscillation frequency of 1 Hz, which is close to the human heart rate (∼1.25 Hz), and at 37 °C (Figure 1A). These values indicate that CH can be safely implanted in the infarcted myocardium, based on the mechanical properties of the different regions of the infarcted heart.^19,20^ The ultimate goal is to not harm the host tissue by implanting an excessively stiff construct.

An adequate cross-linking efficiency was confirmed through free amine quantification by a TNBSA assay for the 3 and 5 mg/mL CH. These values and the resulting polymeric interconnected network strength were expected, as stoichiometric amounts of collagen and 4-arm PEG were added to the reaction mixture in order to fabricate the CH. The 5 mg/mL CH showed an 87% cross-linking efficiency. In comparison, the 3 mg/mL CH had an efficiency of 100%. Steric impediment can explain this behavior, as the increased collagen chain concentration may reduce the availability of the functional groups that are required for covalent cross-linking.^21^ The increased polymer chain density found in the 5 mg/mL samples also increases the storage modulus because it is more difficult for the collagen chains to slide over each other.

SEM micrographs of CH show the expected microstructure,^22^ where pores that are amenable to cell invasion and suitable for drug delivery exist due to the interconnected network that is generated during cross-linking (Figure S1B). Porosity is a key feature that affects the performance of hydrogels for biomedical purposes.^23^ Pore size in particular plays a role in tissue regeneration by enhancing the material’s scaffolding capability and allowing cells to invade and populate the ECM-mimicking matrix and drug diffusion to occur through the water-filled pores.^24^ The estimated porosity and pore size of the fabricated CH decrease as the collagen concentration increases (Table S1). This increased interconnectivity is due to efficient cross-linking and the more compact microstructure that exists when the polymer concentration is higher. Such interconnectivity represents a valuable feature in terms of tissue engineering. Morphologically, the 3 mg/mL CH surface presents an open-pore, though less defined, structure than the 5 mg/mL CH surface, which has a more defined, closed-pore microstructure (Table S1).

Considering that biodegradation of the polymer matrix is one of the key mechanisms for active molecule delivery to the physiological milieu, the CH were tested in the presence of different collagenase concentrations in order to narrow the systems to only those that are able to deliver the cytokine in the time frame of 5–7 days (Figure 1B). The collagenase concentration reported in the literature in an *in vivo* disease scenario is 25 ng/mL.^25^ However, the correlation between *in vitro* and *in vivo* is always uncertain because of the complexity of the *in vivo* micro-environment. In addition, *in vitro* degradation assays are meant to show only the relativity of the degradation response to a control substrate. The final concentration of collagen correlates with the density of the polymer chains that are further cross-linked. As the concentration increases, a more compact mesh is formed, one that is better able to retain active molecules, such as the pro-angiogenic cytokine SDF1. Therefore, by varying the concentration of the polymer matrix, it is possible to modulate the degradation rate and, consequently, the release of therapeutic agents.

Given the mechanical properties and degradation profile of the 3 and 5 mg/mL CH, the highest collagen concentration was chosen for SDF1 loading and for studying the *in vitro* release of SDF1 bioactivity, bCS-SDF1 assembly, and *in vivo* studies.

The collagen-interconnected network constituting the matrix of the hydrogels can efficiently and physically entrap SDF1 molecules and modulate their release into physiological media. SDF1 release from CH (loaded with 100 ng of the cytokine) reached 82% after 120 h of incubation at 37 °C in the presence of 25 ng/mL collagenase in PBS. The released SDF1, quantified by ELISA, depicted a sustained and progressive profile (Figure 1C). In an analogous parallel study that used a collagenase-free incubation medium, only 6% of initially loaded SDF1 was released from the collagen matrix constituting the hydrogels. Together, the outcomes of these two experiments demonstrate the crucial role the matrix biodegradation rate has in governing drug release.

Moreover, it is shown that, in this case, even though a hydrophilic cytokine was accommodated in a hydrophilic matrix, SDF1 diffusion through water-filled pores does not represent a determining release mechanism. The release and dissolution profile of SDF1 from the polymer matrix also show the absence of a burst release. This profile depicts a sustained delivery over 5 days, which relates to the previously described release mechanisms this hydrogel can exert. These results add to the well-supported body of research that has determined CH as suitable candidates for drug delivery systems.

Finally, CH-SDF1 exhibited suitable cytocompatibility over cardiac (HL1 cell line) and ADSC cultures. The putative cytotoxicity of the 5 mg/mL CH over both cell populations was determined by co-incubating CH with the different cell types for 72 and 168 h. CH did not exhibit cytotoxicity over either cell population when compared with the control cells that were cultured without hydrogel exposure (Figure 1D).

**Figure 2:**
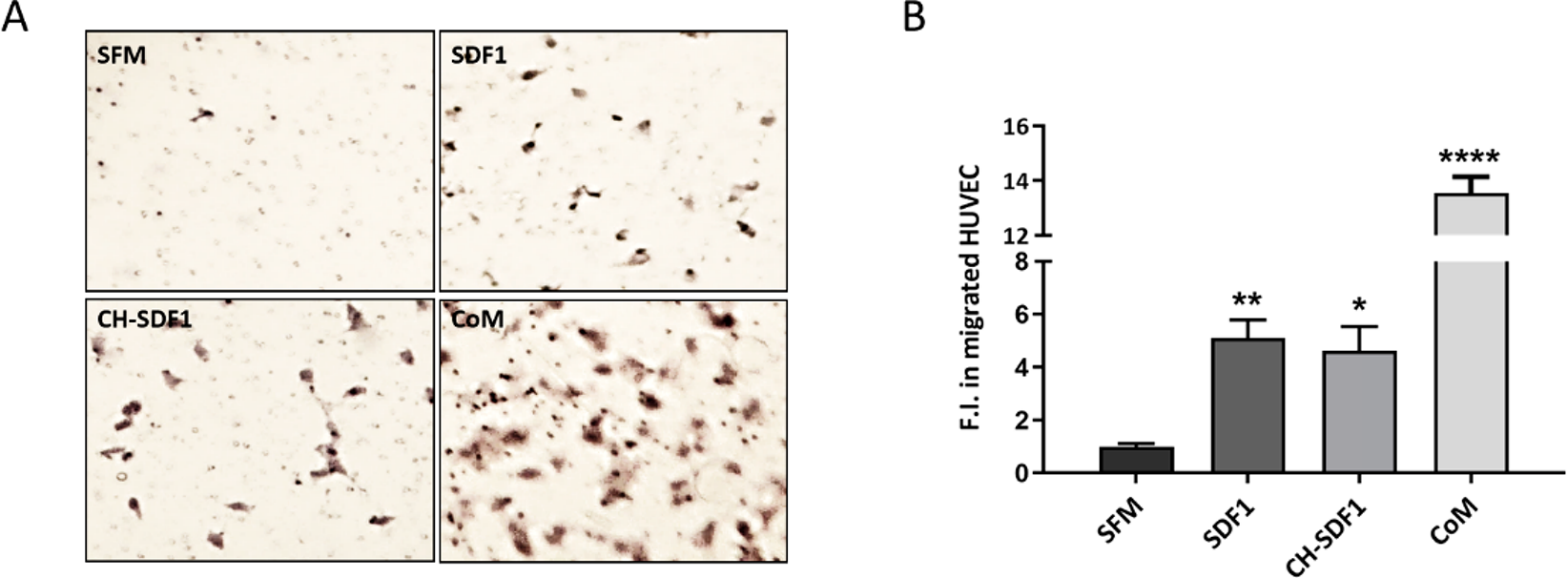
Effect of SDF1 on HUVEC migration *in vitro*. Migrated HUVEC were stained with Harris Hematoxylin in transwell membranes and quantified after 8 h of stimulation with SDF1. (A) Representative pictures that show the migrated cells after being cultured in SFM (negative control), a stimulation medium with free human SDF1 at 20 ng/mL (in SFM), a stimulation medium with human SDF1 released from CH at 20 ng/mL (CH-SDF1), and a CoM (complete medium, positive control). (B) Fold increase in the number of migrated HUVEC in the different experimental groups compared to the nonstimulated cells (SFM = serum-free medium). Three independent experiments were performed in duplicate. Data are represented as mean ± SEM. Statistical significance: *P < 0.05, **P < 0.01, ****P < 0.0001.

The chemoattractant and pro-angiogenic profile of SDF1 recruiting vascular progenitor cells to the injured heart has been extensively proven.^16,17,26^ So, to confirm the bioactivity of SDF1 after its accommodation within collagen-interconnected chains and its subsequent release from the hydrogel matrix, a migration assay was performed on HUVEC cultures in a transwell system. When SDF1 was loaded in the hydrogels, it induced cell migration at a level similar to that of the free cytokine at the same concentration, confirming the bioactivity of the cytokine released from CH-SDF1 (Figure 2).

### 3.2 *In Vivo* Biocompatibility and Functionality of bCS after Implantation in a Rat Subacute MI Model

Two scaffold fabrication technologies were synergistically combined to provide a matrix that is capable of efficiently delivering the therapeutic factor SDF1 to an infarcted myocardium. A compact, nonporous (and, therefore, not suitable for efficient cytokine loading) CM that is able to cater to the required suture strength for surgical implantation^8,9^ was physically coupled in a bilayer pattern with the previously described 5 mg/mL CH, which is able to load and appropriately deliver SDF1 molecules (but is not suitable to provide the required structural support and suture strength on its own). For implantation purposes, 75 μL disc-shaped hydrogels were prepared on top of the CM, thus forming the bCS. The mechanical properties of the bCS were then analyzed. A storage modulus of 2941.7 ± 8.9 Pa and a loss modulus of 2920.0 ± 64.6 Pa were measured at 1 Hz frequency, confirming its suitability for implantation into an infarcted heart.^27^ These patches were sutured to the rat hearts a week after infarct induction, covering the infarct and peri-infarct regions of the ventricle (Figure S2). The hearts were histologically analyzed 7 and 30 days post-implant to study the host compatibility of the implant.

**Figure 3:**
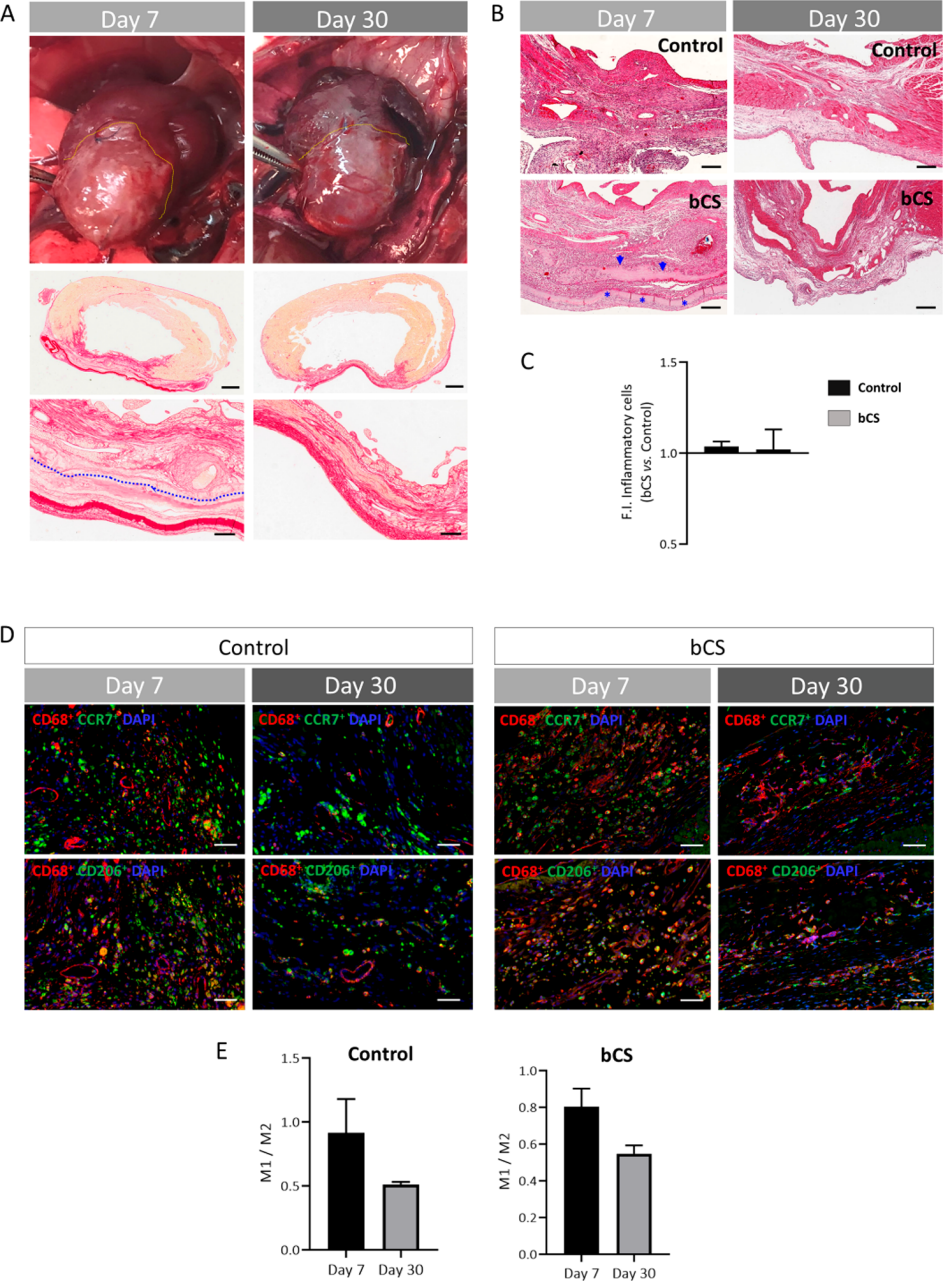
The bCS is biocompatible *in vivo* when implanted in a rat subacute MI model. (A) Representative pictures of hearts that were implanted with bCS taken at 7 and 30 days post-implant, as well as pictures of the Sirius red-stained sections of the hearts. The collagen patch (delineated in yellow) shows good bCS integration into the heart at 7 days post-implant. bCS is practically degraded on day 30. The blue line delimits the bCS from the cardiac tissue. Scale bars: 1 mm (top) and 200 μm (bottom). (B) H&E-stained heart sections at the infarct zones treated with the bCS and not treated with the bCS (control). The blue arrows indicate the presence of the CS, and the asterisks indicate the location of the CH. A strong inflammatory reaction against the bCS in the infarcted hearts is not found at day 7 or day 30 post-implant. Scale bars: 200 μm. (C) The fold increase in the number of inflammatory cells, counted at the infarct zone of the bCS-treated hearts, compared to that of the nontreated ones (control group). (D) Representative immunofluorescence pictures that show the macrophage infiltration in the ischemic heart at the infarct/implant zone (red: anti-CD68, green: anti-CCR7 (M1) or anti-CD206 (M2), and blue: nuclei DAPI staining) in the control and bCS groups. Scale bars: 50 μm. (E) The macrophage M1/M2 ratio at 7 and 30 days after bCS implantation for both the control group (which had no implants performed) and the bCS group (which had implants performed). Data were obtained from 2 to 3 animals per group. Values are represented as mean ± SEM.

Collagen patch integration into the heart was macro- and microscopically analyzed in Sirius red-stained sections. At day 7 post-implant, the scaffolds were macroscopically observed to be covering the hearts, which histologically confirmed their correct adhesion and integration into the heart. Progressive degradation of the patch was also found, and it was scarcely detectable by day 30 post-implant (Figure 3A). Because patch biocompatibility is key for therapeutic use, the putative inflammatory reaction against the bCS was also analyzed at these two time points by quantifying the H&E-stained inflammatory infiltrated cells. Similar levels of inflammation were found between the group implanted with the scaffold and the control group (which was infarcted but not implanted) at day 7 and day 30 post-implant, thus demonstrating the biocompatibility of the patch (Figure 3B,C). Additionally, the phenotype of infiltrated macrophages (pro-inflammatory M1 versus anti-inflammatory M2) was determined at the implant zone. The M1/M2 macrophage ratio was <1 at both time points (as well as in the infarct zone of the control group (the infarcted but not implanted group)), which confirmed the prevalence of anti-inflammatory and pro-angiogenic macrophages in the presence of the collagen-on-collagen patch (Figure 3D).

A similar anti-inflammatory trend was previously observed when we studied the performance of a stand-alone CM after its implantation into heart tissue.^9,28^ Our data are also in agreement with previous studies that have extensively shown the optimal use of collagen for myocardial implantation, further corroborating how collagen matrices can be safely implanted into the heart by showing its mild inflammatory response and its biodegradability capacity, which provide ultimate biocompatibility of the tissue graft.^29−31^ Moreover, the biocompatibility of our particular CM has also been tested in other disease models, such as rat and porcine models of wound healing and urethral structure, which show optimal biocompatibility^32^ and integration into the host tissue with no adverse secondary effects or inflammatory responses.^33,34^

### 3.3 Therapeutic Effect of bCS-SDF1 in a Rat Subacute Infarct Model

**Figure 4:**
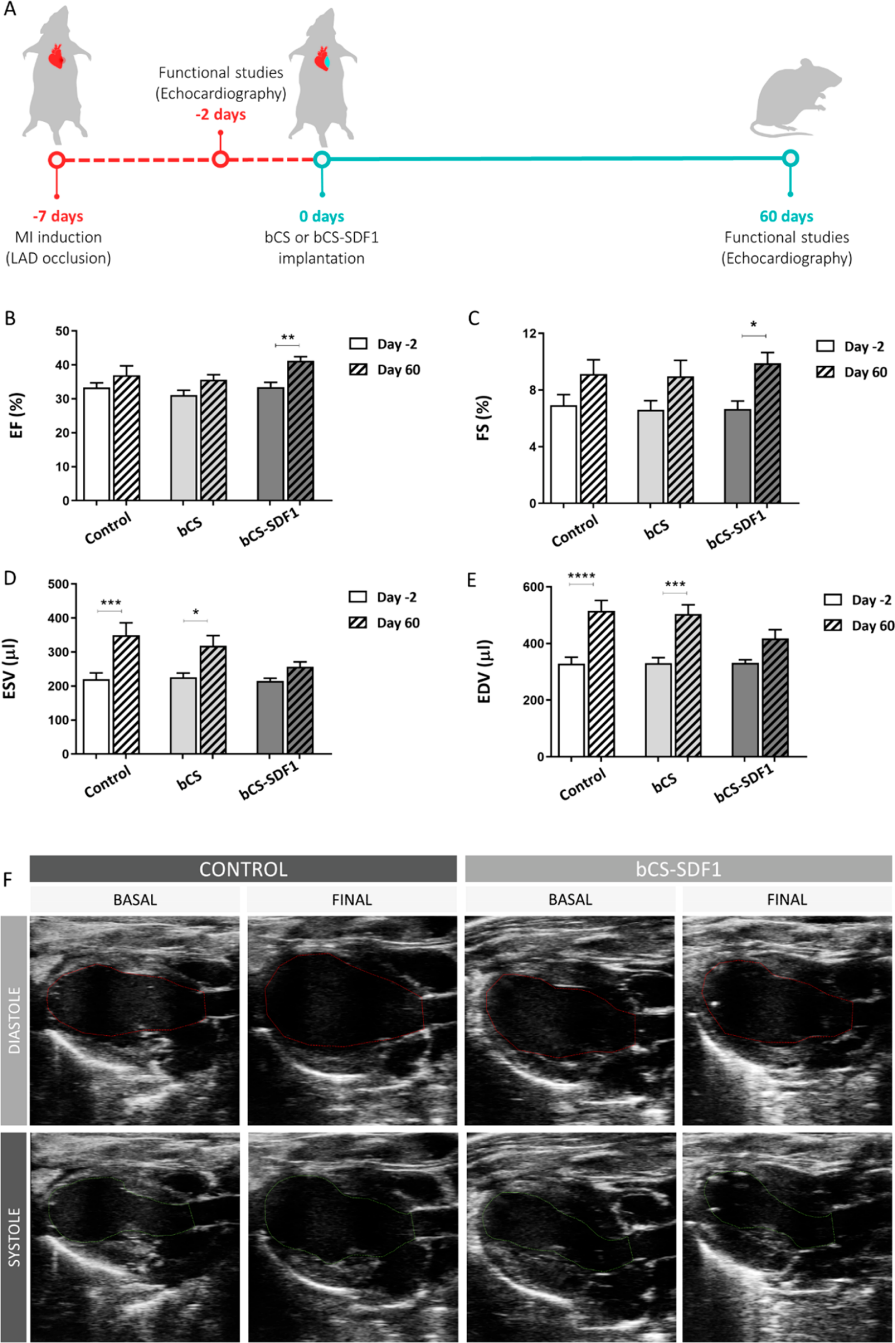
Cardiac functional assessment shows improvement after implantation of bCS-SDF1. (A) The timeline of the experimental procedure. (B) EF (%), (C) FS (%), (D) ESV (μL), and (E) EDV (μL) values measured by echocardiography 5 days post-MI (2 days pre-implant) and 60 days after the implantation of bCS or bCS-SDF1. A group that was operated on but not implanted with bCS was also included as a control. (F) Echocardiographic representative images of the ischemic hearts at diastole and systole. Statistical significance was calculated by ANOVA, with Sidak comparisons between the baseline and the day 60 post-implantation, and is represented as *P < 0.05, **P < 0.01, ***P < 0.001, and ****P < 0.0001. Data were obtained from 7 to 10 animals per group. Mean ± SEM values are represented.

After confirming the biocompatibility of bCS *in vitro* and *in vivo*, the therapeutic effect of bCS-SDF1 was next analyzed in the rat subacute MI model. Cardiac function was assessed by echocardiography 2 days before and 2 months after patch implantation (Figure 4A). By this later point, a significant improvement in the EF and the FS was detected in the animals treated with bCS-SDF1 (Figure 4B,C). Importantly, the deleterious remodeling that was observed in the infarcted hearts, which was evident by a significant increase in the ESV and EDV values (sham and control groups), remained without changing for the ESV and EDV values after treatment with bCS-SDF1. This indicates the beneficial effect of the functionalized patch on heart remodeling after a sustained SDF1 release (Figure 4D–F). Indeed, when the same dose of bolus SDF1 was intramyocardially injected into the heart, no functional benefit was observed (data not shown).

It must be noted that the bCS not loaded with SDF1 did not improve the heart function, as seen in the control group. McLaughlin et al. have recently demonstrated a positive cardiac remodeling in hearts treated at day 7 post-MI with a thermoresponsive recombinant collagen hydrogel matrix (without any added cytokine).^35^ Likewise, other groups have previously demonstrated the biopolymer mechanical support that exists when collagen is acutely injected into the ischemic heart.^36−38^ Collagen patches have also been epicardially delivered to hearts, showing LV-reduced remodeling and neo-vessel formation when treated immediately after infarct induction.^39,40^ The discrepancy between the cited results and our study might be attributed to differences in the delivery strategies and time points. The group of Blackburn et al. has reported that collagen-based matrices confer superior functional benefits when delivered earlier post-MI.^36^ Additionally, the biopolymer features and the delivery route for implantation exert a differential effect, as intramyocardial injection of collagen hydrogels can have a stronger impact on the remodeling of the heart than the delivery through the epicardium.

**Figure 5:**
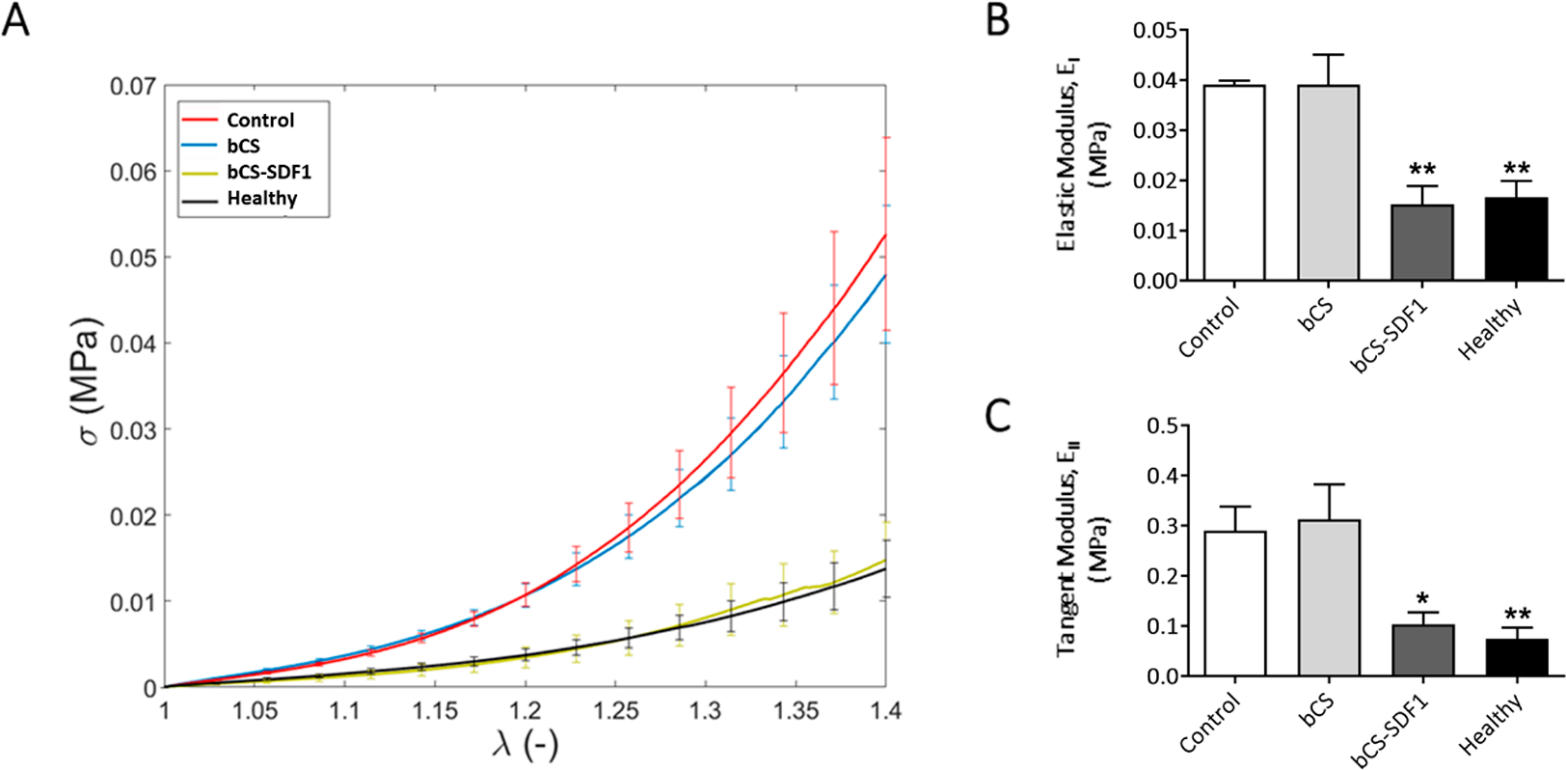
Decreased cardiac tissue stiffness adds to the beneficial effect of bCS-SDF1 treatment after MI. (A) Stress–strain curves for each group. Healthy hearts were included in the analyses (data taken from ref (8)). (B) Elastic and (C) tangential moduli (MPa) of each group. Statistical significance was calculated by ANOVA, with SIDAK comparisons between the control group and the treated and healthy groups at day 60 post-implantation, and is represented as *P < 0.05 and **P < 0.01. Data were obtained from 4 animals per group and represented as mean ± SEM.

As for the mechanical behavior of the ventricular wall, uniaxial tensile tests were performed on heart samples that were implanted with bCS, both loaded and not loaded with SDF1. A non-implanted control group and a healthy group were also included in this analysis. It was found that there were no differences between the control and the nonloaded bCS groups, and both groups showed a significantly stiffer response than the healthy group (Figure 5, Table 1). The heart stiffness in the animals treated with bSC-SDF1 was significantly reduced when compared to the control group and displayed values similar to those with healthy tissue (Figure 5, Table 1). These results are comparable to the biaxial studies that have been performed in rat infarcted hearts that were treated at the acute-injury stage with an SDF1-analogue engineered protein, where native left ventricular mechanical properties were preserved.^44^

**Table 1:** Elastic (*E*_I_) and Tangent (*E*_II_) Moduli^a^

group	*E*_I_ (MPa)	*E*_II_ (MPa)
control	0.039 ± 0.006	0.313 ± 0.070
bCS	0.038 ± 0.001	0.291 ± 0.047
bCS-SDF1	0.015 ± 0.003	0.104 ± 0.022
healthy	0.016 ± 0.003	0.075 ± 0.023)

a Mean ± SEM values are represented. Healthy heart moduli were computed from data taken from ref (8).

**Figure 6:**
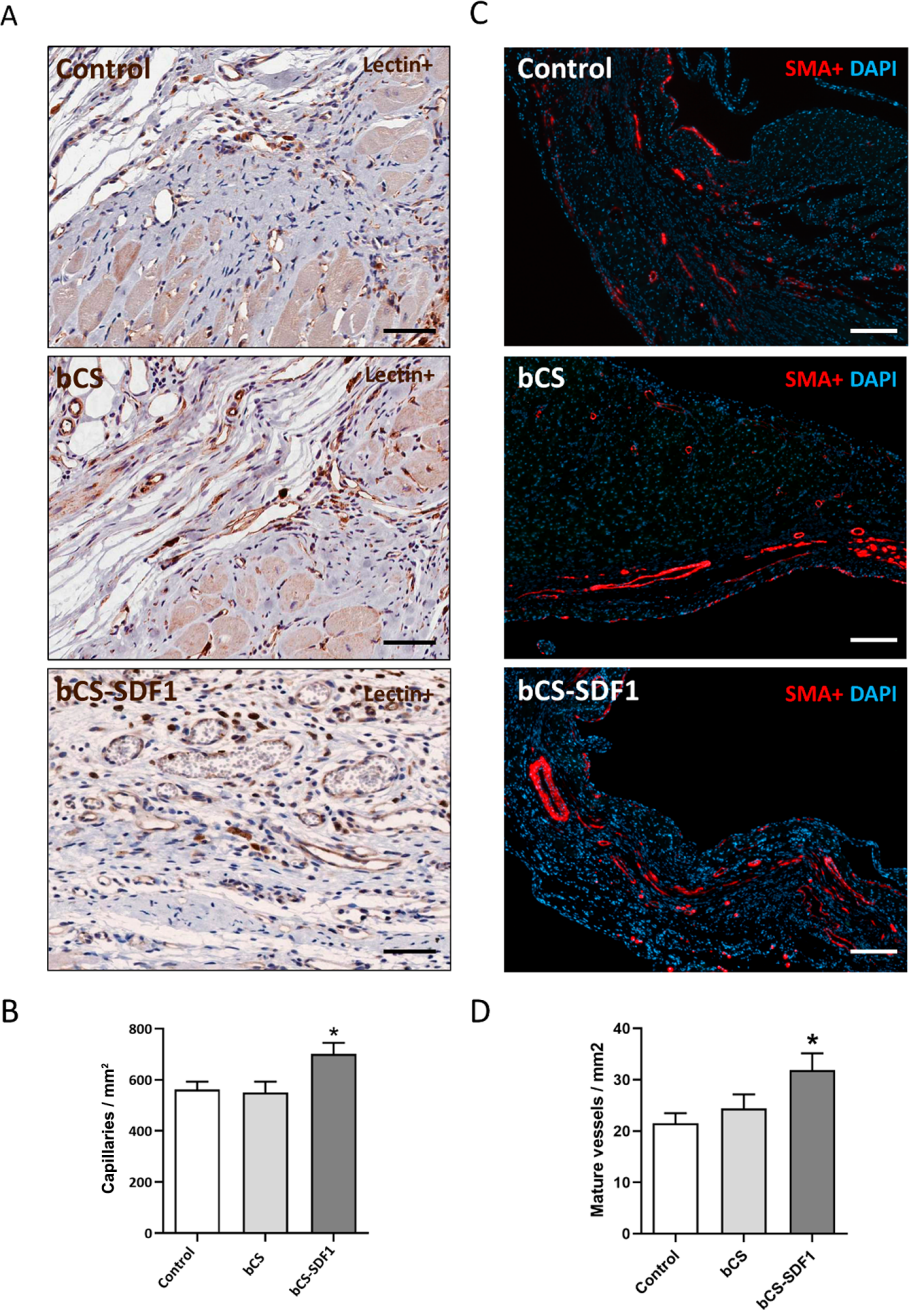
The histological assessment shows increased heart revascularization after bCS-SDF1 implantation. Representative images of (A) the immunohistochemical staining for lectin+ vessels (brown) and (C) the immunofluorescence staining for SMA+ mature vessels (red) at the peri-infarct zone in treated (bCS-SDF1) and nontreated hearts (control and bCS) at day 60 post-MI. Scale bars: 50 μm (lectin staining), 200 μm (SMA staining). (B) Quantified lectin+ capillaries/mm^2^ and (D) SMA+ arterioles-arteries/mm^2^ at the peri-infarct area of the treated and nontreated hearts. Statistical significance was calculated by ANOVA, with Sidak comparisons between the control and treated groups at day 60 post-implantation, and is represented as *P < 0.05. Data were obtained from 7 to 10 animals per group. Mean ± SEM values are represented.

Finally, given the well-known pro-angiogenic role of SDF1,^16,17^ a histological assessment of the hearts’ revascularization was performed in order to elucidate the mechanisms that are involved in the patch-induced functional benefit and positive remodeling. Correlating with this functional improvement, a significant increase in the capillary and mature vessel formation was found in the hearts treated with the SDF1-loaded collagen patch, thus confirming the pro-angiogenic effect of the released cytokine (Figure 6). In addition, a significant induction of apoptosis was not found in the bCS-treated hearts when examined by histological TUNEL staining (data not shown) nor any induction of chronic inflammation when compared with the control group (Figure S3). These results reinforce the concept that the bCS implant can be safely used.

Overall, these functional, mechanical, and histological results demonstrate the therapeutic effect of bCS-SDF1 for the treatment of MI. These data align with previous studies that were performed using different experimental MI models, where the positive effect of the infused or intra(myo)cardially delivered cytokine was shown.^18,19,29^ The use of engineered supra-efficient, pro-angiogenic chemokine analogues^41^ or bifunctional SDF1-AnexinA5 fusion proteins^42^ has rendered encouraging results too. Still, bolus SDF1 bioactivity is rapidly diminished *in vivo* due to the action of myeloid cell-secreted proteases.^43^ Indeed, when the free cytokine was injected, no functional improvement was observed in our case. In that way, our delivery system allows a much more controlled and sustained cytokine release, thus inducing heart repair and avoiding putative secondary adverse effects. Other groups have also developed injectable hydrogels for the sustained delivery of SDF1 to the heart, showing its therapeutic benefits.^44−46^ Interestingly, it is noteworthy that most of these reported studies have assessed the therapeutic potential of SDF1 when administered at the acute stage of the disease (right after infarct induction). In our study, we have determined the benefit of SDF1 when it is sustainedly administered at a subacute stage of the disease (1 week post-infarction in rats), showing that treatment after the initiation of scar formation can also limit pathological cardiac remodeling and promote infarct repair. These results are highly relevant, as treatment at this later stage avoids the high risk of treatment implantation or injection at the acute stage of the infarct and is thus much more translatable to the clinic.

Along with these advantages, our implantable matrix allows for a localized and a much more homogeneous release and distribution of the therapeutic factor to the heart, including the ischemic areas that have impaired perfusion. Furthermore, it is important to consider that, in the physiology of ischemic heart disease, the epicardium always begins to be affected, and ischemia progresses inward toward the endocardium.^47^ If revascularization occurs too early, the endocardium often remains unaffected, which is the case for many ischemic patients that have nontransmural infarcts, who could benefit from epicardial-directed therapy.

Many functionalized acellular patches are currently being tested at the experimental and clinical levels.^48^ A breakthrough occurred with the use of a collagen-based cardiac patch containing follistatin-like 1, which significantly attenuated infarction-induced heart injuries and stimulated the proliferation of endogenous myocytes.^49^ Additionally, the CorMatrix Cor PATCH has been successfully developed by a company. This acellular bioactive material was procured from porcine small intestine submucosa (composed of collagen and proteoglycans, together with bioactive cytokines) and was shown to be compatible with epicardial transplantation. Preclinical and early clinical results support its feasibility and safety and show its clinical benefits.^50^ Consistent with our results, it has been reported that the controlled release of SDF1 via a novel polyethylene glycol-fibrin conjugate patch improves cardiac function in an acute MI model in mice.^51^ Importantly, new approaches for minimally invasive transplantation of the epicardial patches are also being developed in order to reduce the risks and morbidity of the surgical procedure in patients.^52−54^

In summary, we have shown that collagen is a matrix that can act, when appropriately engineered, as a drug delivery system and scaffolding material that promotes functional recovery after MI. Together with this, the scaffold’s host response allows us to use it not only as a cytokine delivery system but also as a cellular carrier. We have previously demonstrated greater engraftment and survival of ADSC into the infarcted heart tissue^8,28^ when implanted with the CM, thus inducing a cardiac functional benefit. Future studies will involve a dual cell-cytokine myocardial collagen patch. In this way, SDF1 may be able to impart a beneficial effect not only in the heart but also in the transplanted mesenchymal cells. The cytoprotective effect of SDF1 on ADSC has been previously proven when cells are exposed to hypoxic and starvation conditions *in vitro* or *in vivo* in a diabetic mouse model of chronic wound healing.^55^ The paracrine, proliferation, and migration abilities of ADSC have also been shown to be simulated after SDF1 treatment.^56^ Additionally, the therapeutic ability of SDF1-pretreated mesenchymal cells has been proven when injected in CTX-induced injured muscles (as evidenced by the fibrosis reduction and inflammatory response modulation that occurs)^57^ and in infarcted myocardium,^58^ where they augment cardiac function and angiogenesis.

Therefore, the acknowledged cytoprotective and stimulating effect of SDF1 toward (stem) cells makes bCS-SDF1 an ideal system that can be seeded with this cell population before its implantation into the heart, thus promoting their survival in the hypoxic environment of the ischemic myocardium.

In the future, our scaffold could also be used as a compliment to the delivery of other types of stem cells, such as induced pluripotent stem cells (iPS), that hold the ability to differentiate into cardiovascular cells;^59^ thus, our scaffold could improve the remodeling of the damaged myocardium by repopulating it with functional cardiomyocytes.

## 4 Conclusions

In this study, a collagen hydrogel, able to load and appropriately deliver pro-angiogenic SDF1, was physically coupled with a compact collagen membrane to provide the suture strength required for surgical implantation. This bilayer collagen-on-collagen scaffold showed suitable physicochemical properties and was proven to be biocompatible and able to be efficiently implanted in an ischemic heart. *In vivo*, its implantation in a rat subacute MI model improved cardiac function 2 months post-MI, which resulted from a significantly increased revascularization and improved cardiac tissue remodeling. Thus, we show that such a scaffold can be tuned for ideal biological and biomechanical properties and can deliver therapeutic biomolecules at the optimal dose rates that are required for appropriate treatment. The proven safety and cost-efficacy advantages of using clinical-grade collagen could favor its easy translation to the clinical scenario.

## Data Availability

The data supporting the findings of this study are available upon request from the corresponding authors.

## Supporting Information Available

Supplemental Figure 1: preparation and SEM characterization of collagen type I hydrogels; Supplemental Figure 2: procedure to fabricate a bCS-SDF1 and *in vivo* implantation in a MI rat model; Supplemental Figure 3: histological assessment of lymphocyte and macrophage infiltration in the heart tissue; and Supplemental Table 1: cross-linking efficiency, associated porosity, and pore size of the studied CH candidates (PDF)

## References

[ref1] Timmis A.; Vardas P.; Townsend N.; Torbica A.; Katus H.; De Smedt D.; Gale C. P; Maggioni A. P; Petersen S. E; Huculeci R.; Kazakiewicz D.; Rubio V. d. B.; Ignatiuk B.; Raisi-Estabragh Z.; Pawlak A.; Karagiannidis E.; Treskes R.; Gaita D.; Beltrame J. F; McConnachie A.; Bardinet I.; Graham I.; Flather M.; Elliott P.; Mossialos E. A; Weidinger F.; Achenbach S. European Society of Cardiology: Cardiovascular Disease Statistics 2021. Eur. Heart J. 2022, 43 (8), 716–799. 10.1093/eurheartj/ehab892. 35016208

[ref2] Frangogiannis N. G. Cardiac Fibrosis. Cardiovasc. Res. 2021, 117 (6), 1450–1488. 10.1093/cvr/cvaa324. 33135058 PMC8152700

[ref3] White S. J.; Chong J. J. H. Growth Factor Therapy for Cardiac Repair: An Overview of Recent Advances and Future Directions. Biophys. Rev. 2020, 12 (4), 805–815. 10.1007/s12551-020-00734-0. 32691300 PMC7429584

[ref4] Arjmand B.; Abedi M.; Arabi M.; Alavi-Moghadam S.; Rezaei-Tavirani M.; Hadavandkhani M.; Tayanloo-Beik A.; Kordi R.; Roudsari P. P.; Larijani B. Regenerative Medicine for the Treatment of Ischemic Heart Disease; Status and Future Perspectives. Front. cell Dev. Biol. 2021, 9, 704903 10.3389/fcell.2021.704903. 34568321 PMC8461329

[ref5] Borrelli M. A.; Turnquist H. R.; Little S. R. Biologics and Their Delivery Systems: Trends in Myocardial Infarction. Adv. Drug Delivery Rev. 2021, 173, 181–215. 10.1016/j.addr.2021.03.014. PMC8178247 33775706

[ref6] Sorushanova A.; Delgado L. M.; Wu Z.; Shologu N.; Kshirsagar A.; Raghunath R.; Mullen A. M.; Bayon Y.; Pandit A.; Raghunath M.; Zeugolis D. I. The Collagen Suprafamily: From Biosynthesis to Advanced Biomaterial Development. Adv. Mater. 2019, 31 (1), 1801651 10.1002/ADMA.201801651. 30126066

[ref7] Chowdhury S. R.; Mh Busra M. F.; Lokanathan Y.; Ng M. H.; Law J. X.; Cletus U. C.; Binti Haji Idrus R. Collagen Type I: A Versatile Biomaterial. Adv. Exp. Med. Biol. 2018, 1077, 389–414. 10.1007/978-981-13-0947-2_21. 30357700

[ref8] Araña M.; Gavira J. J.; Peña E.; González A.; Abizanda G.; Cilla M.; Pérez M. M.; Albiasu E.; Aguado N.; Casado M.; López B.; González S.; Soriano M.; Moreno C.; Merino J.; García-Verdugo J.; Díez J.; Doblaré M.; Pelacho B.; Prosper F. Epicardial Delivery of Collagen Patches with Adipose-Derived Stem Cells in Rat and Minipig Models of Chronic Myocardial Infarction. Biomaterials 2014, 35 (1), 143–151. 10.1016/j.biomaterials.2013.09.083. 24119456

[ref9] Araña M.; Peña E.; Abizanda G.; Cilla M.; Ochoa I.; Gavira J. J.; Espinosa G.; Doblaré M.; Pelacho B.; Prosper F. Preparation and Characterization of Collagen-Based ADSC-Carrier Sheets for Cardiovascular Application. Acta Biomater. 2013, 9 (4), 6075–6083. 10.1016/j.actbio.2012.12.014. 23261927

[ref10] Meyer M. Processing of Collagen Based Biomaterials and the Resulting Materials Properties. Biomed. Eng. Online 2019, 18 (1), 1–74. 10.1186/s12938-019-0647-0. 30885217 PMC6423854

[ref11] Figuereido I.; Paiotta A.; Dal Magro R.; Tinelli F.; Corti R.; Re F.; Cassina V.; Caneva E.; Nicotra F.; Russo L. A New Approach for Glyco-Functionalization of Collagen-Based Biomaterials. Int. J. Mol. Sci. 2019, 20 (7), 1747 10.3390/IJMS20071747. 30970594 PMC6480084

[ref12] Yamauchi M.; Sricholpech M. Lysine Post-Translational Modifications of Collagen. Essays Biochem. 2012, 52 (1), 113–133. 10.1042/bse0520113. 22708567 PMC3499978

[ref13] Rebelo A. L.; Chevalier M. T.; Russo L.; Pandit A. Sweet Tailoring of Glyco-Modulatory Extracellular Matrix-Inspired Biomaterials to Target Neuroinflammation. Cell Reports Phys. Sci. 2021, 2 (2), 100321 10.1016/j.xcrp.2021.100321.

[ref14] Sampaolesi S.; Nicotra F.; Russo L. Glycans in Nanomedicine, Impact and Perspectives. Future Med. Chem. 2019, 11 (1), 43–60. 10.4155/fmc-2018-0368. 30526037

[ref15] Rebelo A. L.; Bizeau J.; Russo L.; Pandit A. Glycan-Functionalized Collagen Hydrogels Modulate the Glycoenvironment of a Neuronal Primary Culture. Biomacromolecules 2020, 21 (7), 2681–2694. 10.1021/acs.biomac.0c00387. 32433878

[ref16] Ghadge S. K.; Mühlstedt S.; Özcelik C.; Bader M. SDF-1α as a Therapeutic Stem Cell Homing Factor in Myocardial Infarction. Pharmacol. Ther. 2011, 129 (1), 97–108. 10.1016/j.pharmthera.2010.09.011. 20965212

[ref17] Li J. H.; Li Y.; Huang D.; Yao M. Role of Stromal Cell-Derived Factor-1 in Endothelial Progenitor Cell-Mediated Vascular Repair and Regeneration. Tissue Eng. Regen. Med. 2021, 18 (5), 747–758. 10.1007/s13770-021-00366-9. 34449064 PMC8440704

[ref18] Venugopal J. R.; Prabhakaran M. P.; Mukherjee S.; Ravichandran R.; Dan K.; Ramakrishna S. Biomaterial Strategies for Alleviation of Myocardial Infarction. J. R. Soc. Interface 2012, 9 (66), 1–19. 10.1098/rsif.2011.0301. 21900319 PMC3223634

[ref19] Hiesinger W.; Brukman M. J.; McCormick R. C.; Fitzpatrick J. R.; Frederick J. R.; Yang E. C.; Muenzer J. R.; Marotta N. A.; Berry M. F.; Atluri P.; Woo Y. J. Myocardial Tissue Elastic Properties Determined by Atomic Force Microscopy after Stromal Cell-Derived Factor 1α Angiogenic Therapy for Acute Myocardial Infarction in a Murine Model. J. Thorac. Cardiovasc. Surg. 2012, 143 (4), 962–966. 10.1016/j.jtcvs.2011.12.028. 22264415 PMC4155937

[ref20] Pislaru C.; Urban M. W.; Pislaru S. V.; Kinnick R. R.; Greenleaf J. F. Viscoelastic Properties of Normal and Infarcted Myocardium Measured by a Multifrequency Shear Wave Method: Comparison with Pressure-Segment Length Method. Ultrasound Med. Biol. 2014, 40 (8), 1785–1795. 10.1016/j.ultrasmedbio.2014.03.004. 24814645 PMC4118646

[ref21] Monaghan M.; Browne S.; Schenke-Layland K.; Pandit A. A Collagen-Based Scaffold Delivering Exogenous Microrna-29B to Modulate Extracellular Matrix Remodeling. Mol. Ther. 2014, 22 (4), 786–796. 10.1038/mt.2013.288. 24402185 PMC3983959

[ref22] He Y.; Wang C.; Wang C.; Xiao Y.; Lin W. An Overview on Collagen and Gelatin-Based Cryogels: Fabrication, Classification, Properties and Biomedical Applications. Polymers (Basel). 2021, 13 (14), 2299 10.3390/polym13142299. 34301056 PMC8309424

[ref23] Annabi N.; Nichol J. W.; Zhong X.; Ji C.; Koshy S.; Khademhosseini A.; Dehghani F. Controlling the Porosity and Microarchitecture of Hydrogels for Tissue Engineering. Tissue Eng. Part B. Rev. 2010, 16 (4), 371–383. 10.1089/ten.teb.2009.0639. 20121414 PMC2946907

[ref24] González De Torre I.; Santos M.; Quintanilla L.; Testera A.; Alonso M.; Rodríguez Cabello J. C. Elastin-like Recombinamer Catalyst-Free Click Gels: Characterization of Poroelastic and Intrinsic Viscoelastic Properties. Acta Biomater. 2014, 10 (6), 2495–2505. 10.1016/j.actbio.2014.02.006. 24530853

[ref25] Fertin M.; Lemesle G.; Turkieh A.; Beseme O.; Chwastyniak M.; Amouyel P.; Bauters C.; Pinet F. Serum MMP-8: A Novel Indicator of Left Ventricular Remodeling and Cardiac Outcome in Patients after Acute Myocardial Infarction. PLoS One 2013, 8 (8), e71280 10.1371/JOURNAL.PONE.0071280. 23967183 PMC3743841

[ref26] Cheng M.; Huang K.; Zhou J.; Yan D.; Tang Y. L.; Zhao T. C.; Miller R. J.; Kishore R.; Losordo D. W.; Qin G. A Critical Role of Src Family Kinase in SDF-1/CXCR4-Mediated Bone-Marrow Progenitor Cell Recruitment to the Ischemic Heart. J. Mol. Cell. Cardiol. 2015, 81, 49–53. 10.1016/j.yjmcc.2015.01.024. 25655934 PMC4380859

[ref27] Emig R.; Zgierski-Johnston C. M.; Timmermann V.; Taberner A. J.; Nash M. P.; Kohl P.; Peyronnet R. Passive Myocardial Mechanical Properties: Meaning, Measurement, Models. Biophys. Rev. 2021, 13 (5), 587–610. 10.1007/s12551-021-00838-1. 34765043 PMC8555034

[ref28] Castellano D.; Blanes M.; Marco B.; Cerrada I.; Ruiz-Saurí A.; Pelacho B.; Araña M.; Montero J. A.; Cambra V.; Prosper F.; Sepúlveda P. A Comparison of Electrospun Polymers Reveals Poly(3-Hydroxybutyrate) Fiber as a Superior Scaffold for Cardiac Repair. Stem Cells Dev. 2014, 23 (13), 1479–1490. 10.1089/scd.2013.0578. 24564648 PMC4066229

[ref29] Gao J.; Liu J.; Gao Y.; Wang C.; Zhao Y.; Chen B.; Xiao Z.; Miao Q.; Dai J. A Myocardial Patch Made of Collagen Membranes Loaded with Collagen-Binding Human Vascular Endothelial Growth Factor Accelerates Healing of the Injured Rabbit Heart. Tissue Eng. Part A 2011, 17 (21–22), 2739–2747. 10.1089/ten.tea.2011.0105. 21682575

[ref30] Miyagi Y.; Chiu L. L. Y.; Cimini M.; Weisel R. D.; Radisic M.; Li R. K. Biodegradable Collagen Patch with Covalently Immobilized VEGF for Myocardial Repair. Biomaterials 2011, 32 (5), 1280–1290. 10.1016/j.biomaterials.2010.10.007. 21035179

[ref31] Holladay C. A.; Duffy A. M.; Chen X.; Sefton M. V.; O’Brien T. D.; Pandit A. S. Recovery of Cardiac Function Mediated by MSC and Interleukin-10 Plasmid Functionalised Scaffold. Biomaterials 2012, 33 (5), 1303–1314. 10.1016/j.biomaterials.2011.10.019. 22078809

[ref32] Rahmanian-Schwarz A.; Held M.; Knoeller T.; Stachon S.; Schmidt T.; Schaller H. E.; Just L. In Vivo Biocompatibility and Biodegradation of a Novel Thin and Mechanically Stable Collagen Scaffold. J. Biomed. Mater. Res., Part A 2014, 102 (4), 1173–1179. 10.1002/jbm.a.34793. 23666868

[ref33] Aufderklamm S.; Vaegler M.; Kelp A.; Maurer S.; Gustafsson L.; Mundhenk J.; Busch S.; Daum L.; Stenzl A.; Amend B.; Sievert K. D. Collagen Cell Carriers Seeded with Human Urothelial Cells for Urethral Reconstructive Surgery: First Results in a Xenograft Minipig Model. World J. Urol. 2017, 35 (7), 1125–1132. 10.1007/s00345-016-1959-3. 27783146

[ref34] Sievert K. D.; Daum L.; Maurer S.; Toomey P.; Vaegler M.; Aufderklamm S.; Amend B. Urethroplasty Performed with an Autologous Urothelium-Vegetated Collagen Fleece to Treat Urethral Stricture in the Minipig Model. World J. Urol. 2020, 38 (9), 2123–2131. 10.1007/s00345-019-02888-3. 31502031

[ref35] McLaughlin S.; McNeill B.; Podrebarac J.; Hosoyama K.; Sedlakova V.; Cron G.; Smyth D.; Seymour R.; Goel K.; Liang W.; Rayner K. J.; Ruel M.; Suuronen E. J.; Alarcon E. I. Injectable Human Recombinant Collagen Matrices Limit Adverse Remodeling and Improve Cardiac Function after Myocardial Infarction. Nat. Commun. 2019, 10 (1), 4866 10.1038/s41467-019-12748-8. 31653830 PMC6814728

[ref36] Blackburn N. J. R.; Sofrenovic T.; Kuraitis D.; Ahmadi A.; McNeill B.; Deng C.; Rayner K. J.; Zhong Z.; Ruel M.; Suuronen E. J. Timing Underpins the Benefits Associated with Injectable Collagen Biomaterial Therapy for the Treatment of Myocardial Infarction. Biomaterials 2015, 39, 182–192. 10.1016/j.biomaterials.2014.11.004. 25468370

[ref37] Neuta P. A.; Rojas D. M.; Agredo W.; Gutierrez J. O. Evaluation of the Repairing Effect of Collagen Type I and MaxGel on the Infarcted Myocardium in an Animal Model. IEEE Eng. Med. Biol. Soc. Annu. Int. Conf. 2015, 3529–3532. 10.1109/EMBC.2015.7319154. 26737054

[ref38] Dai W.; Wold L. E.; Dow J. S.; Kloner R. A. Thickening of the Infarcted Wall by Collagen Injection Improves Left Ventricular Function in Rats: A Novel Approach to Preserve Cardiac Function after Myocardial Infarction. J. Am. Coll. Cardiol. 2005, 46 (4), 714–719. 10.1016/j.jacc.2005.04.056. 16098441

[ref39] Serpooshan V.; Zhao M.; Metzler S. A.; Wei K.; Shah P. B.; Wang A.; Mahmoudi M.; Malkovskiy A. V.; Rajadas J.; Butte M. J.; Bernstein D.; Ruiz-Lozano P. The Effect of Bioengineered Acellular Collagen Patch on Cardiac Remodeling and Ventricular Function Post Myocardial Infarction. Biomaterials 2013, 34 (36), 9048–9055. 10.1016/j.biomaterials.2013.08.017. 23992980 PMC3809823

[ref40] Gaballa M. A.; Sunkomat J. N. E.; Thai H.; Morkin E.; Ewy G.; Goldman S. Grafting an Acellular 3-Dimensional Collagen Scaffold onto a Non-Transmural Infarcted Myocardium Induces Neo-Angiogenesis and Reduces Cardiac Remodeling. J. Heart Lung Transplant. 2006, 25 (8), 946–954. 10.1016/j.healun.2006.04.008. 16890116

[ref41] Wang H.; Wisneski A.; Paulsen M. J.; Imbrie-Moore A.; Wang Z.; Xuan Y.; Hernandez H. L.; Lucian H. J.; Eskandari A.; Thakore A. D.; Farry J. M.; Hironaka C. E.; von Bornstaedt D.; Steele A. N.; Stapleton L. M.; Williams K. M.; Wu M. A.; MacArthur J. W.; Woo Y. J. Bioengineered Analog of Stromal Cell-Derived Factor 1α Preserves the Biaxial Mechanical Properties of Native Myocardium after Infarction. J. Mech. Behav. Biomed. Mater. 2019, 96, 165–171. 10.1016/j.jmbbm.2019.04.014. 31035067 PMC6701187

[ref42] Huang F. Y.; Xia T. L.; Li J. L.; Li C. M.; Zhao Z. G.; Lei W. H.; Chen L.; Liao Y. B.; Xiao D.; Peng Y.; Wang Y. B.; Liu X. J.; Chen M. The Bifunctional SDF-1-AnxA5 Fusion Protein Protects Cardiac Function after Myocardial Infarction. J. Cell. Mol. Med. 2019, 23 (11), 7673–7684. 10.1111/jcmm.14640. 31468674 PMC6815779

[ref43] Valenzuela-Fernández A.; Planchenault T.; Baleux F.; Staropoli I.; Le-Barillec K.; Leduc D.; Delaunay T.; Lazarini F.; Virelizier J. L.; Chignard M.; Pidard D.; Arenzana-Seisdedos F. Leukocyte Elastase Negatively Regulates Stromal Cell-Derived Factor-1 (SDF-1)/CXCR4 Binding and Functions by Amino-Terminal Processing of SDF-1 and CXCR4. J. Biol. Chem. 2002, 277 (18), 15677–15689. 10.1074/jbc.M111388200. 11867624

[ref44] Ding Y.; Zhao A.-S.; Liu T.; Wang Y.-N.; Gao Y.; Li J. an; Yang P. An Injectable Nanocomposite Hydrogel for Potential Application of Vascularization and Tissue Repair. Ann. Biomed. Eng. 2020, 48 (5), 1511–1523. 10.1007/S10439-020-02471-7. 32034609

[ref45] Efraim Y.; Sarig H.; Cohen Anavy N.; Sarig U.; de Berardinis E.; Chaw S. Y.; Krishnamoorthi M.; Kalifa J.; Bogireddi H.; Duc T. V.; Kofidis T.; Baruch L.; Boey F. Y. C.; Venkatraman S. S.; Machluf M. Biohybrid Cardiac ECM-Based Hydrogels Improve Long Term Cardiac Function Post Myocardial Infarction. Acta Biomater. 2017, 50, 220–233. 10.1016/j.actbio.2016.12.015. 27956366

[ref46] Macarthur J. W.; Purcell B. P.; Shudo Y.; Cohen J. E.; Fairman A.; Trubelja A.; Patel J.; Hsiao P.; Yang E.; Lloyd K.; Hiesinger W.; Atluri P.; Burdick J. A.; Woo Y. J. Sustained Release of Engineered Stromal Cell-Derived Factor 1-α from Injectable Hydrogels Effectively Recruits Endothelial Progenitor Cells and Preserves Ventricular Function after Myocardial Infarction. Circulation 2013, 128, S79–S86. 10.1161/CIRCULATIONAHA.112.000343. 24030424 PMC4111255

[ref47] Frangogiannis N. G. Pathophysiology of Myocardial Infarction. Compr. Physiol. 2015, 5 (4), 1841–1875. 10.1002/cphy.c150006. 26426469

[ref48] Vasanthan V.; Fatehi Hassanabad A.; Pattar S.; Niklewski P.; Wagner K.; Fedak P. W. M. Promoting Cardiac Regeneration and Repair Using Acellular Biomaterials. Front. Bioeng. Biotechnol. 2020, 8, 291 10.3389/fbioe.2020.00291. 32363184 PMC7180212

[ref49] Wei K.; Serpooshan V.; Hurtado C.; Diez-Cunado M.; Zhao M.; Maruyama S.; Zhu W.; Fajardo G.; Noseda M.; Nakamura K.; Tian X.; Liu Q.; Wang A.; Matsuura Y.; Bushway P.; Cai W.; Savchenko A.; Mahmoudi M.; Schneider M. D.; Van Den Hoff M. J. B.; Butte M. J.; Yang P. C.; Walsh K.; Zhou B.; Bernstein D.; Mercola M.; Ruiz-Lozano P. Epicardial FSTL1 Reconstitution Regenerates the Adult Mammalian Heart. Nature 2015, 525 (7570), 479–485. 10.1038/nature15372. 26375005 PMC4762253

[ref50] Vasanthan V.; Biglioli M.; Hassanabad A. F.; Dundas J.; Matheny R. G.; Fedak P. W. M. The CorMatrix Cor^TM^ PATCH for Epicardial Infarct Repair. Future Cardiol. 2021, 17 (8), 1297–1305. 10.2217/fca-2021-0017. 34008420

[ref51] Zhang G.; Nakamura Y.; Wang X.; Hu Q.; Suggs L. J.; Zhang J. Controlled Release of Stromal Cell-Derived Factor-1 Alpha in Situ Increases c-Kit+ Cell Homing to the Infarcted Heart. Tissue Eng. 2007, 13 (8), 2063–2071. 10.1089/ten.2006.0013. 17518719

[ref52] Tedde M. L.; Brito Filho F.; Belmonte E. D. A.; Pinto Filho D. R.; Pereira S. T. F. L.; Okumura E. M.; Melani A. G. F.; Gossot D. Video-Assisted Thoracoscopic Surgery in Swine: An Animal Model for Thoracoscopic Lobectomy Training. Interact. Cardiovasc. Thorac. Surg. 2015, 21 (2), 224–230. 10.1093/icvts/ivv138. 26015506

[ref53] Cattaneo S. M.; Park B. J.; Wilton A. S.; Seshan V. E.; Bains M. S.; Downey R. J.; Flores R. M.; Rizk N.; Rusch V. W. Use of Video-Assisted Thoracic Surgery for Lobectomy in the Elderly Results in Fewer Complications. Ann. Thorac. Surg. 2008, 85 (1), 231–236. 10.1016/j.athoracsur.2007.07.080. 18154816

[ref54] Hazelrigg S. R.; Mack M. J.; Landreneau R. J.; Acuff T. E.; Seifert P. E.; Auer J. E. Thoracoscopic Pericardiectomy for Effusive Pericardial Disease. Ann. Thorac. Surg. 1993, 56 (3), 792–795. 10.1016/0003-4975(93)90982-N. 8379795

[ref55] Li Q.; Guo Y.; Chen F.; Liu J.; Jin P. Stromal Cell-Derived Factor-1 Promotes Human Adipose Tissue-Derived Stem Cell Survival and Chronic Wound Healing. Exp. Ther. Med. 2016, 12 (1), 45–50. 10.3892/etm.2016.3309. 27347016 PMC4906949

[ref56] Li Q.; Zhang A.; Tao C.; Li X.; Jin P. The Role of SDF-1-CXCR4/CXCR7 Axis in Biological Behaviors of Adipose Tissue-Derived Mesenchymal Stem Cells in Vitro. Biochem. Biophys. Res. Commun. 2013, 441 (3), 675–680. 10.1016/j.bbrc.2013.10.071. 24184476

[ref57] Zimowska M.; Archacka K.; Brzoska E.; Bem J.; Czerwinska A. M.; Grabowska I.; Kasprzycka P.; Michalczewska E.; Stepaniec I.; Soszynska M.; Ilach K.; Streminska W.; Ciemerych M. A. IL-4 and SDF-1 Increase Adipose Tissue-Derived Stromal Cell Ability to Improve Rat Skeletal Muscle Regeneration. Int. J. Mol. Sci. 2020, 21 (9), 3302 10.3390/ijms21093302. 32392778 PMC7246596

[ref58] Esmaeili R.; Darbandi-Azar A.; Sadeghpour A.; Majidzadeh-A K.; Eini L.; Jafarbeik-Iravani N.; Hoseinpour P.; Vajhi A.; Oghabi Bakhshaiesh T.; Masoudkabir F.; Sadeghizadeh M. Mesenchymal Stem Cells Pretreatment With Stromal-Derived Factor-1 Alpha Augments Cardiac Function and Angiogenesis in Infarcted Myocardium. Am. J. Med. Sci. 2021, 361 (6), 765–775. 10.1016/j.amjms.2021.01.025. 33582157

[ref59] Mei X.; Cheng K. Recent Development in Therapeutic Cardiac Patches. Front. Cardiovasc. Med. 2020, 7, 610364 10.3389/fcvm.2020.610364. 33330673 PMC7728668

