# Supplementary material for: A Multimodal Scaffold for SDF1 Delivery Improves Cardiac Function in a Rat Subacute Myocardial Infarct Model
Source: ACS Appl Mater Interfaces. 2023 Aug 11;15(44):50638–51. doi: 10.1021/acsami.3c04245 (PMC10636708; doi:10.1021/acsami.3c04245)
Supplement: Supplementary file 1 — am3c04245_si_001.pdf [file am3c04245_si_001.pdf]

Supplementary Information

A multimodal scaffold for SDF-1 delivery improves cardiac function in a rat subacute myocardial infarct model

Iñigo Perez-Estenaga [1#], Merari Chevalier [2#], Estefania Pena [3,4], Gloria Abizanda [1,5], Amir M Alsharabasy [2], Eduardo Larequi [1], Myriam Cilla [3,4], Marta Perez [6], Jon Gurtubay [1], Manuel Garcia-Yebenes Castro [7], Felipe Prosper [1,5,8,9] Abhay Pandit [2*], Beatriz Pelacho [1,5*]

[1]Regenerative Medicine Department, Center for Applied Medical Research (CIMA), University of Navarra, Pamplona, Spain, [2] CÚRAM, SFI Research Center for Medical Devices University of, Galway; Ireland; [3]Aragon Institute of Engineering Research, University of Zaragoza, Zaragoza, Spain; [4]CIBER-BBN-Centro de Investigación Biomédica en Red en Bioingeniería Biomateriales y Nanomedicina, Zaragoza, Spain. [5]Instituto de Investigación Sanitaria de Navarra (IdiSNA), Pamplona, Spain. [6]Department of Anatomy, Embryology and Genetics, University of Zaragoza, Spain. [7]Department of Cardiology, Clínica Universidad de Navarra, Spain. [8]Department of Cell Therapy and Hematology, Clínica Universidad de Navarra, Spain. [9]CIBERONC, Spain

[#] These authors share the first authorship

*** These authors share the last authorship and are corresponding authors**

Beatriz Pelacho: bpelacho@unav.es

Abhay Pandit: abhay.pandit@nuigalway.ie

Supplemental Figures

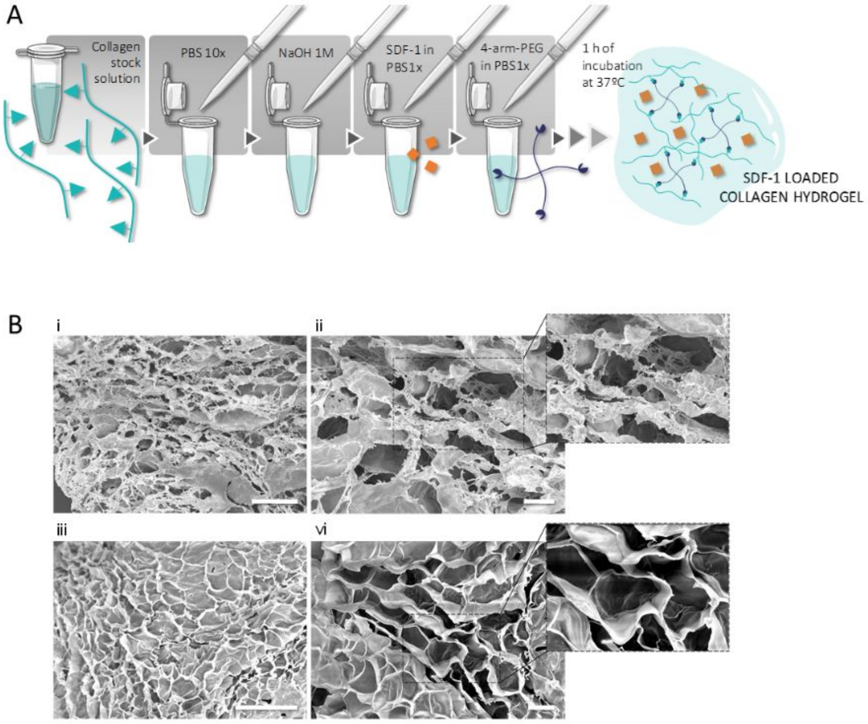

Supplemental Figure 1. **Preparation and SEM characterization of collagen type I hydrogels. A)** Fabrication of CH-SDF1. Hydrogels were fabricated through an optimized protocol of covalent crosslinking collagen primary amino groups and NHS ester reactive groups from 4arm-PEG-Succinimidyl Glutarate crosslinker. SDF1 molecules are physically entrapped within the matrix interconnected network to be subsequently released. **B)** Representative SEM micrographs. 3 mg/mL (i and ii) and 5 mg/mL (iii and iv) hydrogels are displayed. ii and iv exhibit more detailed magnifications showing pore morphology and microstructure integrity. 5 mg/mL hydrogel presents a more defined and closed-pore microstructure than the 3 mg/mL hydrogel open-pore and less dense pore distribution. Scale bars: 50 μm (i and iii), 10μm (ii and iv).

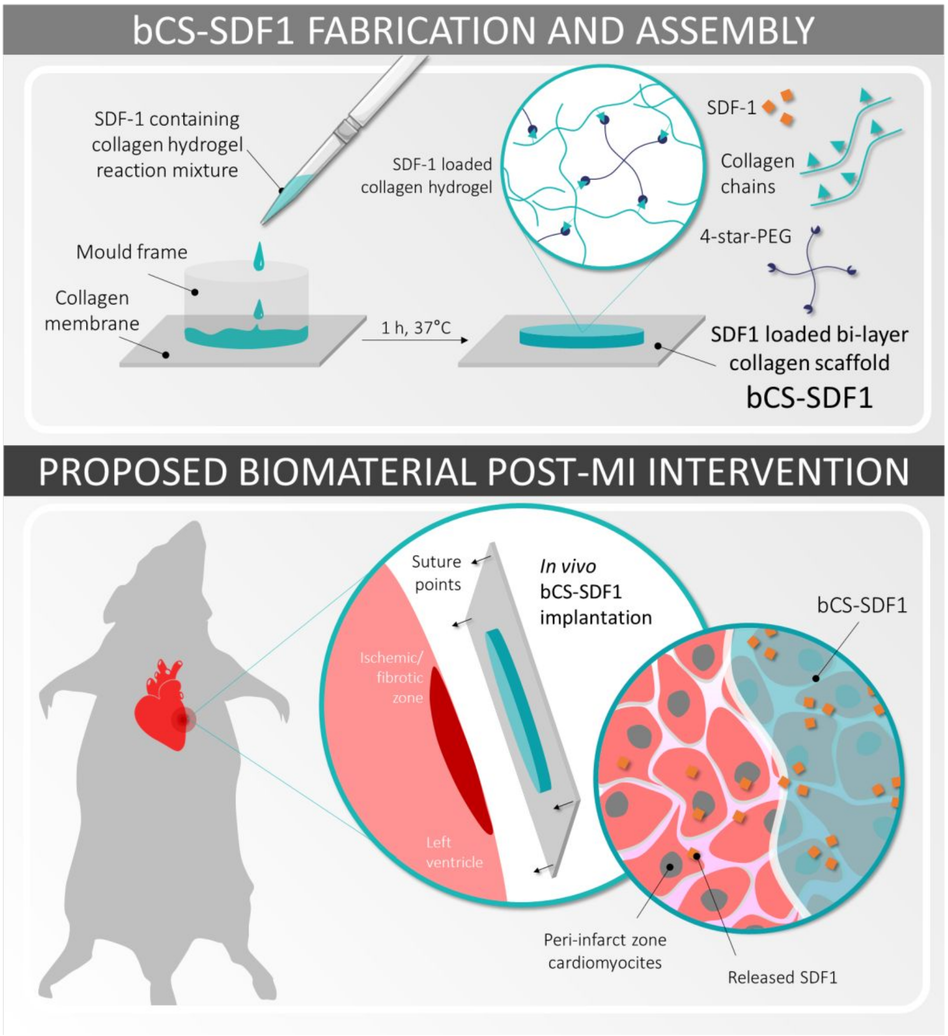

Supplementary Figure 2. Procedure to fabricate a bCS-SDF1 and *in vivo* implantation in a MI rat model. Top box: A framed mould is used to prepare disc-shaped hydrogels assembled by adhesion onto collagen membranes and render a bCS able to deliver SDF1. Down box: The fabricated bCS-SDF1 is implanted into the pericardial wall of the myocardium by suturing the collagen scaffold at four points with the collagen hydrogel layer in contact with the damaged cardiac tissue.

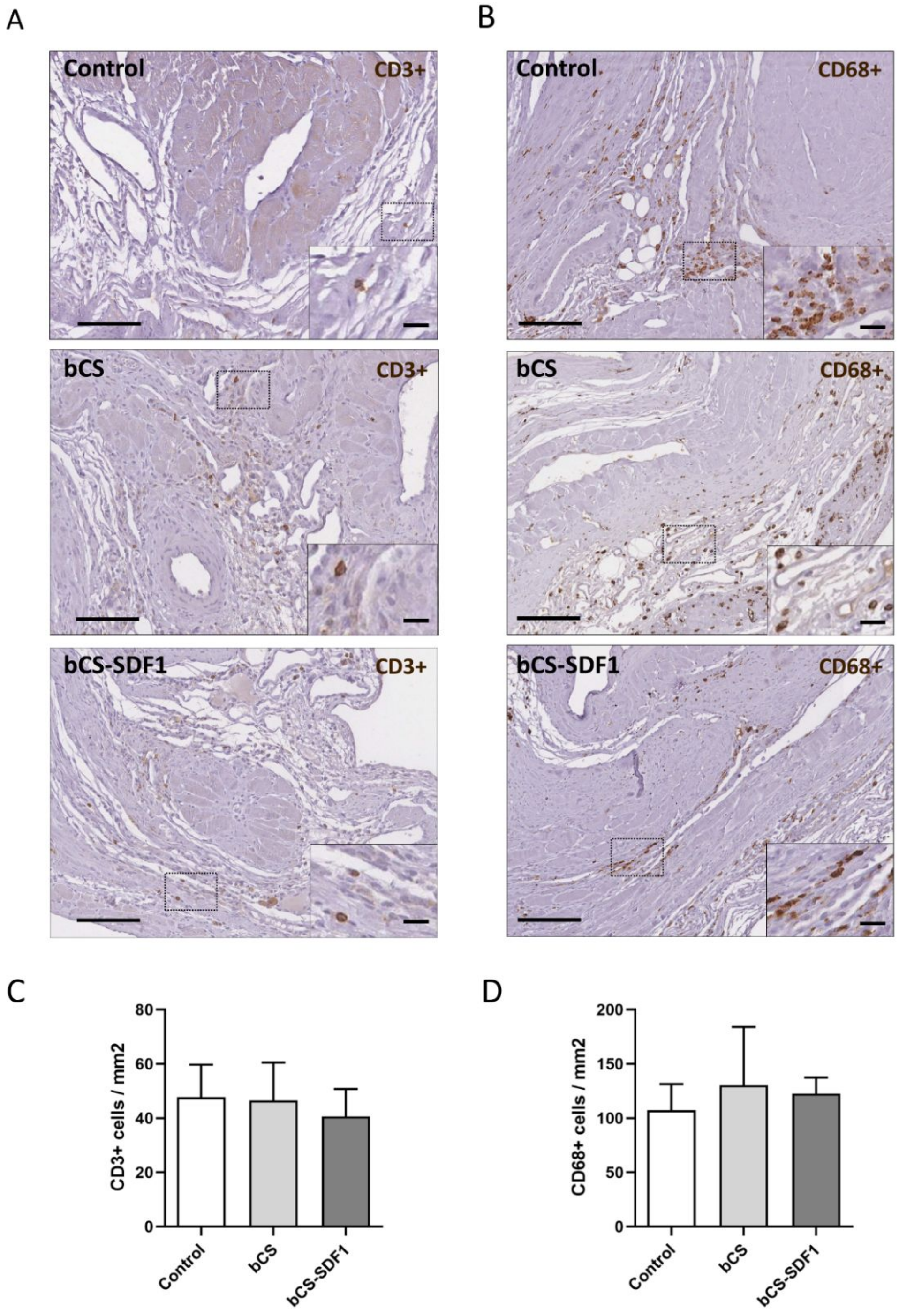

Supplementary Figure 3. Histological assessment of lymphocyte and macrophage infiltration in the heart tissue. Representative images of immunohistochemical staining for CD3+ **(A)** and CD68+ cells **(B)** at the peri-infarct zones in treated (bCS-SDF1) and non-treated hearts (Control and bCS) at day 60 post-MI. Scale bars: 100 μm. Quantified lymphocytes and macrophages/mm^2 at the peri-infarct region of the treated and non-treated hearts **(C, D)**. Statistical significance is calculated by ANOVA with SIDAK comparisons between the control and treated groups at day 60 post-implantation. No significant differences were found in comparison with the control group. Data were obtained from 4-5 animals per group. Mean ± SEM values are represented.

Supplemental Table 1. Crosslinking efficiency, associated porosity, and pore size of the studied CH candidates. Analyses were performed in triplicate. Mean ± SEM values are represented.

Collagen concentration (mg/mL)	Ratio Collagen: 4-arm-PEG	Crosslinking efficiency (%)	Porosity (%)	Pore size (µm)
3	1:1	100±3	78.98 ± 1.63	25.86 ± 1.2
5	1:1	87±13	72.56 ± 1.73	11.88 ± 0.5
